# Non-Invasive Determination of the Paternal Inheritance in Pregnancies at Risk for β-Thalassaemia by Analyzing Cell-Free Fetal DNA Using Targeted Next-Generation Sequencing

**DOI:** 10.3390/ijms26020570

**Published:** 2025-01-10

**Authors:** Stefania Byrou, Rutger W. W. Brouwer, Marios Tomazou, Stella Tamana, Petros Kountouris, Carsten W. Lederer, Miranda Petrou, Zeliha Ozgur, Xander den Dekker, Zakia Azmani, Soteroula Christou, Christiana Makariou, Marina Kleanthous, Wilfred F. J. van IJcken, Thessalia Papasavva

**Affiliations:** 1 Molecular Genetics Thalassaemia Department, The Cyprus Institute of Neurology & Genetics, Nicosia 2371, Cyprus; stefaniab@cing.ac.cy (S.B.); stellat@cing.ac.cy (S.T.); petrosk@cing.ac.cy (P.K.); lederer@cing.ac.cy (C.W.L.); miranda@cing.ac.cy (M.P.); marinakl@cing.ac.cy (M.K.); 2 Department of Cell Biology, Erasmus University Medical Center, P.O. Box 2040, 3000 CA Rotterdam, The Netherlands; rutger.brouwer@futurefacts.nl (R.W.W.B.); z.ozgur@erasmusmc.nl (Z.O.); a.dendekker@erasmusmc.nl (X.d.D.); z.azmani@erasmusmc.nl (Z.A.); 3 Center for Biomics, Erasmus University Medical Center, P.O. Box 2040, 3000 CA Rotterdam, The Netherlands; 4 Nicosia Thalassaemia Clinic, Archbishop Makarios III Hospital, Nicosia 2371, Cyprus; snchrthalcl@cytanet.com.cy; 5 Thalassaemia Screening Laboratory, Thalassaemia Center, Archbishop Makarios III Hospital, Nicosia 2371, Cyprus; chmakariou@gmail.com

**Keywords:** non-invasive prenatal testing (NIPT), β-thalassaemia, cell-free DNA (cfDNA), haplotype analysis, monogenic disorders

## Abstract

Non-invasive prenatal testing (NIPT) has been widely adopted for the screening of chromosomal abnormalities; however, its adoption for monogenic disorders, such as β-thalassaemia, has proven challenging. Haemoglobinopathies are the most common monogenic disorders globally, with β-thalassaemia being particularly prevalent in Cyprus. This study introduces a non-invasive prenatal haplotyping (NIPH) assay for β-thalassaemia, utilizing cell-free DNA (cfDNA) from maternal plasma. The assay determines paternal inheritance by analyzing highly heterozygous single-nucleotide variants (SNVs) in the β-globin gene cluster. To identify highly heterozygous SNVs in the population, 96 randomly selected samples were processed using Illumina DNA-prep NGS chemistry. A custom, high-density NGS genotyping panel, named HAPLONID, was designed with 169 SNVs, including 15 common pathogenic ones. The AmpliSeq for Illumina assay was then applied to cfDNA to evaluate the panel’s efficiency in performing NIPT for β-thalassaemia. Analysis revealed 219 highly polymorphic SNVs, and the sequencing of 17 families confirmed successful paternal allele determination. The NIPH assay demonstrated 100% success in diagnostic interpretation. This study achieved the advancement of an integrated NGS-NIPT assay for β-thalassaemia, bringing it one step closer to being a diagnostic assay and thereby enabling a reduction in the number of risky invasive prenatal sampling procedures in Cyprus and elsewhere.

## 1. Introduction

The discovery of degraded, non-encapsulated DNA fragments within blood plasma, also termed cell-free DNA (cfDNA), triggered further investigation of its potential use as a diagnostic marker. To this end, in 1997, the presence of fetal genetic material was discovered in maternal blood [1], providing an invaluable tool for non-invasive prenatal testing (NIPT). Cell-free fetal DNA (cffDNA) is randomly fragmented in short sizes and represents approximately 3−20% [2,3] of total cfDNA in maternal circulation. 

The prenatal diagnostic methods that have been used for decades are amniocentesis (AC) and chorionic villi sampling (CVS), which are invasive and provide diagnostic results around the 16th and 12th week of pregnancy, respectively. Both AC and CVS carry risks such as infection, preterm labor, amniotic fluid leak, and potential fetal injury, with miscarriage rates estimated at around 1.2% for amniocentesis and 0.7% for CVS [4]. Therefore, switching from invasive to non-invasive procedures can be beneficial in multiple ways, combining safe sampling with earlier diagnostic inferences (~8th week of gestation) and thus reducing parental anxiety and distress. 

Recently, NIPT for fetal chromosomal aneuploidy testing [5,6,7], fetal sex determination [8,9,10], and assessments of fetal Rhesus status [11,12] has progressed from research to clinical practice. Different methods and approaches have also been used by several groups for the development of an NIPT assay for single-gene disorders [13,14,15,16]. However, this has proven to be technically challenging as maternal and fetal genomes share homology and are only being differentiated by single-point variant differences. 

Early NIPT efforts for monogenic disorders were focused on the detection or exclusion of the paternally inherited fetal pathogenic variant based on the exclusive presence of paternal SNVs in fetal as opposed to maternal DNA in the maternal plasma [17,18,19,20,21,22]. Nevertheless, these approaches are limited to autosomal dominant disorders or to compound heterozygosity in autosomal recessive diseases. Therefore, in populations of low genetic diversity and the frequent occurrence of homozygosity for autosomal recessive disorders, such as in Cyprus, this approach is frequently unsuitable for couples at risk. 

In Cyprus, β-thalassaemia, an autosomal recessive single-gene disorder caused by pathogenic variants located on the *HBB* gene [23], is of particular prevalence. Statistics show an approximate 12% population-wide carrier frequency for β-thalassaemia [24], of which 79% carry the intronic NM_000518.5:c.[93-21G>A] β-globin pathogenic variant [25]. If left untreated, β-thalassaemia can be lethal. Therefore, the early diagnosis of the disease is essential for its proper management. 

Up until now, a plethora of methodologies have been used for the NIPT of β-thalassaemia, including real-time PCR [26,27], droplet digital PCR (ddPCR) [14,28] and next-generation sequencing (NGS, also known as massively parallel sequencing) [29,30]. In 2013, our group published successful NIPT implementation in 8 families at risk for β-thalassaemia out of the 10 examined by analyzing four single-nucleotide variants (SNVs) via targeted NGS [13]. Furthermore, Vermeulen et al. showed the feasibility of NIPT for β-thalassaemia and two other monogenic disorders using NGS and Targeted Locus Amplification (TLA) for direct parental haplotype phasing [31]. In addition, our team recently showed that the ddPCR-based detection of maternal inheritance coupled with relative mutation dosage (RMD) is also possible when targeting known β-thalassaemia pathogenic variants [14]. 

The extensive research work performed for the NIPT of single-gene disorders gave rise to successful approaches, but the majority of them are stalled at the proof-of-concept stage and do not reach clinical practice. In general, approaches based on single-point analysis are unsuitable, because they are highly prone to misdiagnosis, whereas for autosomal recessive diseases, many approaches are limited to couples who carry different pathogenic variants. 

In a previous study, we presented Fast Temperature-Gradient COLD PCR technology developed in-house, which has been optimized for two SNVs for the NIPT of β-haemoglobinopathies. This assay can successfully detect the paternally inherited fetal SNVs for haplotype analysis implementation, thus promoting non-invasive diagnosis, even in cases where parents share the same pathogenic variant [15]. However, the study also emphasizes the need to analyze a higher number of SNVs, rather than to rely on a small set, to achieve more accurate fetal haplotype inference through high-throughput methodologies, such as NGS.

The current study expands the number of targeted variants and combines the examination of the primary pathogenic variants of the HBB gene with the analysis of a large number of SNVs located on the β-globin gene cluster through targeted NGS for direct and indirect result inference, respectively. The resulting approach equally facilitates haplotype analysis implementation and prenatal diagnosis deduction, thus reducing the risk of misdiagnosis and providing high diagnostic accuracy and specificity. 

The analysis of a large number of polymorphic variants enables the inference of parental inheritance through haplotype analysis. This also works for couples who share the same pathogenic variant. This is of particular importance in Cyprus, where most of the couples share the same β-thalassaemia pathogenic variant [25]. In addition, this approach provides more reliable and universal NIPT analysis than the use of single-point pathogenic variants alone and thus overcomes the necessity to develop tailor-made tests on a family basis.

**Figure 1:**
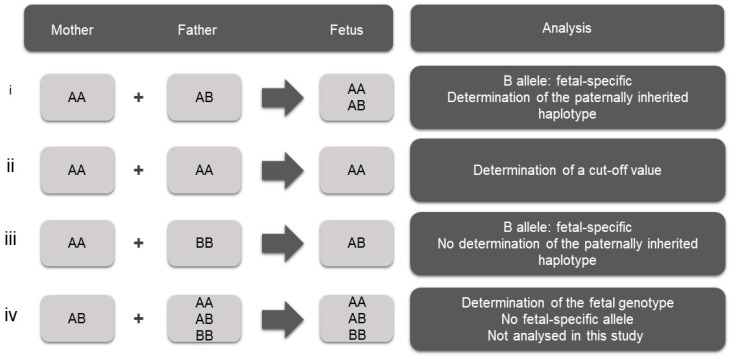
Parental SNV combinations separated into four different groups for NIPT analysis. (**i**) The determination of the paternally inherited fetal haplotype using SNVs for which the mother is homozygous and the father is heterozygous, enabling the detection of the fetal-specific allele that is absent from the maternal genome. (**ii**) The determination of the cut-off value of the assay, using SNVs for which both parents are homozygous for the same allele. (**iii**) The confirmation of the presence of fetal DNA in maternal plasma using SNVs for which both parents are homozygous but for different alleles. (**iv**) The determination of the fetal genotype using SNVs for which the mother is heterozygous.

The criterion for the selection of SNVs is a high degree of heterozygosity, rendering them informative in haplotype analysis and parental inheritance inference. Informative SNVs are those for which the mother is homozygous and the father is heterozygous, enabling the detection of the fetal-specific allele that is absent from the maternal genome and thus the determination of the paternally inherited fetal haplotype (Figure 1i). This selection of homozygous variants for the mother leaves the phase of the maternal alleles undetermined. SNVs for which both parents are homozygous for the same allele were also used in this study for the determination of the cut-off value of the assay (Figure 1ii). SNVs for which both parents are homozygous for a different allele can also be used to confirm the presence of fetal DNA in maternal plasma (Figure 1iii). This scenario was not observed in the selected SNVs in the present study and therefore was not used. In addition, SNVs for which the mother is heterozygous can be used for the determination of the fetal genotype (Figure 1iv). However, this approach requires advanced and highly sophisticated bioinformatics analysis, as there is no fetal-specific allele; therefore, differentiation between maternal and fetal alleles is extremely challenging. This approach was not applied in this study.

The objectives of this study are (i) the identification of the high-heterozygosity variants located on the β-globin gene cluster in the Greek–Cypriot population, (ii) the design of a custom, high-density NGS panel of oligonucleotides that targets the HBB gene and the pre-determined, highly heterozygous variants, (iii) the assessment of the sensitivity and specificity of the custom-designed targeted NGS assay in determining paternal inheritance via the analysis of cfDNA samples and (iv) the evaluation of the feasibility of non-invasive prenatal haplotype analysis of β-thalassaemia using the genomic DNA (gDNA) of the partners and the CVS sample simultaneously with the corresponding cfDNA sample.

## 2. Results

### 2.1. Identification of High-Heterozygosity SNVs

In order to maximize the possibility of having a high number of informative SNVs for haplotype NIPT analysis, the β-globin gene cluster variants that exhibit a high degree of heterozygosity in the Cypriot population should be targeted. In order to determine those, 96 randomly selected Greek–Cypriot individuals were analyzed using the Illumina DNA-prep library preparation kit. Variants for which more than 95% of the population had the homozygous reference allele were considered common, encompassing a total of 324 out of 660 variants. From the remaining 336 variants, 271 SNVs were identified with a degree of heterozygosity between 10% and 90%, while 65 SNVs with values above 90% were excluded since they did not follow the Mendelian inheritance pattern. For the determination and subsequent rejection of all SNVs that did not follow the Mendelian inheritance, the Hardy–Weinberg Equilibrium was used. Overall, 219 SNVs out of the 271 had a degree of heterozygosity between 10 and 90% and a P-value higher than 0.05 (Supplementary Materials, Table S1). These 219 SNVs were selected to be targeted together with the HBB gene for the development of a reliable NIPT assay for β-thalassaemia using targeted NGS. 

### 2.2. High-Density Custom-Designed Genotyping Panel Haplonid

Because our NIPT approach is based on the analysis of multiple SNVs for the inference of the fetal inheritance, a custom-made panel was designed to target the pre-defined highly heterozygous SNVs and the HBB gene. 

After evaluating and optimizing the design, the 15 β-thalassaemia mutations most common in Cyprus [25] were covered, along with the 80 most common β-thalassaemia pathogenic variants globally (Table S2). Furthermore, the final design captured 154 out of the 219 highly heterozygous SNVs (~70%) (Table S1), with approximately 81% of the total targeted bases being covered by the design.

One hundred and fifty-seven amplicons were designed in total, with fragment length ranging from 40 to 154 bp and an average amplicon size of 76 bp, excluding primers. Primers were estimated to be approximately 23 bp each.

### 2.3. Sensitivity Determination of the High-Density Custom-Designed Genotyping Panel

The analysis of 17 cfDNA samples from pregnancies at risk for β-thalassaemia was performed for the pathogenic variants of each family and the 154 pre-selected SNVs located on the β-globin gene cluster using our custom genotyping panel. In order to test the detection limit of the assay and its sensitivity, artificial mixtures of two gDNA samples with low concentrations were created, and variable proportions of one sample were spiked into the other, i.e., 1.25%, 2.5%, 5%, and 10% (Section 4.7). Informative SNVs, i.e., SNVs for which the gDNA being in excess was homozygous while the gDNA in minority was heterozygous, were identified to calculate the fraction of the minor genotype within the spiked mixture (Table S3). The calculations were based on Equation (1) (Section 4.9), and a situation with an allele being in the minority was considered as a simulation of the fetal-specific allele. A statistical analysis was then performed for each spiked sample (Table 1). It was observed that the expected fractions of the minor genotype in the 1.25%, 2.5%, and 10% spiked samples fell within the range of the mean value ± 1 standard deviation (SD). However, despite the expected fraction of the 5% spiked sample lying outside the window of the mean value ± 1 SD, the distance from the maximum value of 4.58% was insignificant (i.e., 0.42%). Therefore, these findings demonstrate the sensitivity and the efficiency of the assay for detecting and differentiating the minor allele in an overwhelming background of a major allele.

### 2.4. Determination of a Cut-Off Value

In order to assess the efficiency of the assay for paternally inherited fetal allele detection in cfDNA, a cutoff value needs to be determined for the prevention of false result inference. For this purpose, a selection was made of SNVs, for which both parents in each family share the same homozygosity (Figure 1ii), given that it is biologically impossible for a different allele to be present in the cfDNA samples of those families. Subsequently, the calculation of the error rate was performed for each SNV (Table S4) based on Equation (1) (Section 4.9) and the maximum error rate value was determined per family and cfDNA replicate. 

We empirically compared the true and false detection rates of the fetal allele by adding either 1 or 2 SDs to the maximum error rate per family for the first nine families analyzed. The results showed minimal differences between these two approaches; however, detection rates were slightly improved when 1 SD was added (Table S5). Based on this observation, we adopted the approach of adding 1 SD to establish the cutoff value, above which a positive call for fetal allele detection was made (Table 2).

### 2.5. Detection of Paternally Inherited Fetal Allele

To assess the sensitivity and specificity of the assay for the true positive and true negative detection of the paternally inherited fetal-specific allele in cfDNA, respectively, the analysis of informative SNVs, i.e., where the mother is homozygous and the father is heterozygous, was implemented (Figure 1i). A total of 125 informative SNVs were identified for paternally inherited fetal allele detection in this study among the 17 families examined (Table S6). Significant variability was observed in the number of informative SNVs between the 17 families, as is illustrated in Table 3.

**Figure 2:**
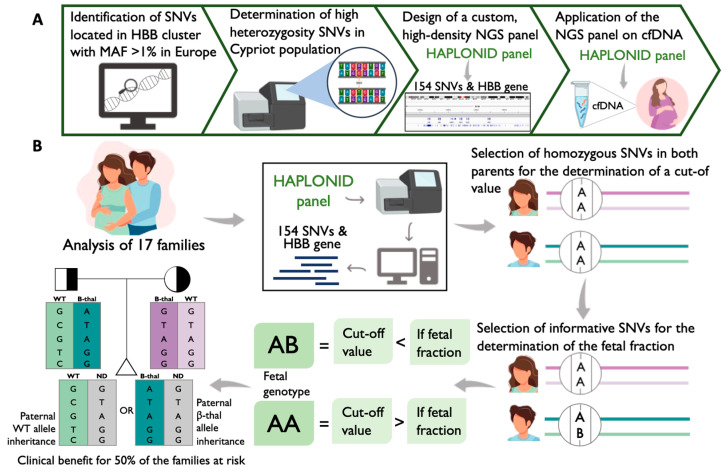
A schematic overview of the study. (**A**) A four-step process involving in silico identification of SNVs in the β-globin cluster, the determination of highly heterozygous SNVs in the Cypriot population, and the design of the HAPLONID NGS panel as well as its application to cfDNA samples. (**B**) The analysis of 17 families using the HAPLONID panel. Homozygous SNVs in both parents were selected to establish a family-specific cut-off value, while informative SNVs (where the mother is homozygous and the father is heterozygous) were used to determine the fetal fraction. If the fetal fraction exceeded the cut-off, it was inferred that the fetus had inherited the paternal fetal-specific allele B; if it was below the cut-off, the non-fetal-specific allele A was inferred. Fetal haplotypes were then constructed to provide diagnosis. In cases where the paternal wild-type (WT) allele was inherited, non-invasive prenatal diagnosis (NIPD) could characterize the fetus as either a carrier or as unaffected by β-thalassaemia. Conversely, if the paternal β-thal allele was inherited, the fetal status remained uncertain, precluding NIPD. This approach proved clinically beneficial for 50% of at-risk families.

The cfDNA analysis results were compared with the CVS DNA genotyping results, as CVS enables a direct analysis of the fetal sample, whereas cfDNA is a mixture of maternal and fetal DNA. In the present study, a true positive case refers to an informative SNV where the fetal-specific paternal allele is correctly identified in the cfDNA sample, with the fetal fraction exceeding the cut-off value for that specific sample (as detailed in Table 2). Conversely, a true negative case refers to an informative SNV where the fetal-specific paternal allele is correctly identified as absent in the cfDNA sample, with the fetal fraction falling below the cut-off value set for that sample (Figure 2B) (Table S7). Upon comparison with the CVS DNA, the cffDNA genotype call was successful in 453 out of the 481 cases, of which 9 were the paternal pathogenic variants of families 1, 8, 10, 11, 13, 15 and 17. In these families, the parents shared different pathogenic variants; therefore, these were informative for our analyses. More specifically, the direct detection of the paternally inherited pathogenic allele was accomplished with a fetal fraction of 2.76% for replicate 275_a and a fraction of 4.46% for replicate 275_b in family 1, a 6.38% fraction in family 10, a 2.57% fraction in family 11, a 8.16% fraction for the NM_000518.5:c.[92+6T>C] pathogenic variant in family 13, and a 3.59% fraction for family 17. At the same time, the direct negative detection of the paternally inherited pathogenic allele was achieved with a fetal fraction of 0.07% in family 8, a fraction of 0% for the NM_000518.5:c.[-137C>G] pathogenic variant in family 13, and a fraction of 0.11% in family 15 (Table S7). The remaining 26 out of the 481 cases represent minimal discordance between CVS and cfDNA analysis (Table S7). 

Moreover, regarding families 1–4, for which two cfDNA replicates per family were analyzed, considerable variation was observed in the number of true positive results between the two replicates of family 1, with the one being 64% and the other 82% (Table 4). Nonetheless, our analysis yielded consistent outcomes between the duplicates of families 2, 3, and 4, with 100% true positive (families 2 and 4) and true negative (families 2 and 3) detection. 

A true positive detection of the paternal allele was accomplished in 51/67 informative SNVs (76%) for family 5, in 29/30 SNVs (97%) for family 15, and in 24/26 SNVs (92%) for family 17, while a true negative detection of the paternal allele in 5/6 informative SNVs (83%) was achieved for family 10 and in 68/70 (97%) for family 11. Significantly, the completely successful true positive (families 6, 8, 12, and 13) and true negative (families 7, 8, 9, 13, and 16) detection of the fetal allele at 100% was achieved in families 6, 7, 8, 9, 12, 13, and 16. Importantly, minimal false-positive detection was observed in 1/6 informative SNVs (17%) in family 10 and in 2/70 SNVs (3%) in family 11. Furthermore, false-negative detection of the paternal allele was only observed, in families 1, 5, 15, and 17 in 4/11 (36%), 2/11 (18%), 16/67 (24%), 1/30 (3%), and in 2/26 (8%) informative SNVs, respectively. The true and false detection rates obtained from the present study are illustrated in Table 4. 

The specificity, sensitivity, and rate of false-positive and false-negative fetal allele detection were determined for each informative SNV based on the comparison of the cfDNA analysis with the CVS analysis. Significantly, the assay’s specificity was 98.8% (95% CI: 96.64–99.76), the sensitivity was 88.8% (95% CI: 83.9–92.6), while the percentage values of the false-positive and false-negative fetal allele detection rate were 1.1% (95% CI: 0.24–3.36) and 11.2% (95% CI: 7.39–16.1), respectively. These findings indicate the high specificity of the assay in detecting and differentiating the paternally inherited fetal allele. However, more optimization is needed to reduce the number of false-negative results and increase the assay’s sensitivity.

### 2.6. Non-Invasive Fetal Haplotyping

In order to examine the possibility of using SNVs to detect paternal inheritance in the NIPT of β-thalassaemia, haplotype analysis was implemented for 16 out of the 17 cfDNA samples tested, as there were no informative SNVs for one of the families analyzed.

Determining the paternal inheritance in each cfDNA tested presupposes determination of the phase of the parental alleles. The determination of the phase of the paternal alleles in each family was based on the informative SNVs in conjunction with the pathogenic variant in cases where the parents shared different pathogenic variants. The phase of the maternal alleles in each family was determined based on the pathogenic variant alone, since the analyzed SNVs were pre-selected to be homozygous for the mother. As a consequence, and because the pathogenic variant of the mother belonged to group iv of Figure 1, for which no analysis was performed, the phase of the maternally inherited fetal alleles in the cfDNA samples could not be determined. 

In families where the fetus was homozygous, β-thalassaemia-affected (i.e., families 1, 3, 10, 11 and 13), or homozygous wild-type-non-affected (i.e., family 8 and 15), the phasing of the parental alleles was based on the CVS DNA genotyping results obtained from the NGS analysis. On the other hand, the determination of the phase of the parental alleles for families 4, 5, 6, 7, 9, and 12 was based on previous family studies performed in our laboratory, since the parents and the fetus were carriers of the same pathogenic variant. The phasing of the parental alleles in families 2, 16, and 17 was determined based on the NGS analysis of the grandparents, representing a real-case scenario. 

After parental haplotype phasing, the fetal haplotypes were determined for 16 out of the 17 families examined based on cfDNA-NGS analysis (i.e., families 1–13 and 15–17). Haplotype analysis was not applicable for family 14, since there were no informative SNVs for this family. 

The inferred NIPT analysis for families 2, 3, 4, 6, 7, 8, 9, 12, 13, and 16 was based on all the informative SNVs analyzed for each cffDNA, demonstrating 100% concordance with CVS analysis. The NIPT for family 1 was based on 7 out of the 11 analyzed variants for the first replicate (sample ID: 275_a) and on 9 out of the 11 variants for the second replicate (sample ID:275_b), while for family 5, the NIPT relied on 51 out of the 67 informative SNVs analyzed. The concordance with the CVS haplotypes in these cases was 64%, 82%, and 76%, respectively, and since it was higher than 50%, it allowed the unambiguous determination of the paternally inherited fetal haplotype. For the same reason, the determination of the paternally inherited fetal haplotype was also allowed for families 10, 11, 15, and 17. The NIPT for family 10 was based on 13 out of 13 true positive variants (100%) and 5 out of 6 true negative variants (83%), while the NIPT for family 11 was based on 1 out of 1 true positive variant (100%) and 68 out of 70 true negative variants (97%). Regarding family 15, the NIPT result was based on 29 out of 30 true positive variants (97%) and 1 out of 1 true negative variant (100%), while for family 17, it was based on 24 out of 26 true positive variants (92%) and 1 out of 1 true negative variant (100%). Significantly, the final non-invasive prenatal diagnosis regarding the paternally inherited fetal allele was successful for all the 16 families examined based on NIPH, despite the minimal false-positive and false-negative detection rates observed.

For families 2, 4, 5, 6, 8, 12, 15, and 16, where the fetus inherited the wild-type paternal allele and therefore was predicted to be non-affected, either as β-thalassaemia carrier or with absence of the β-thalassaemia-associated allele, the need for invasive prenatal diagnosis was avoided (Table S8). Furthermore, in families 1, 3, 7, 9, 10, 11, 13, and 17, where the fetus was predicted to have inherited the β-thalassaemia allele of the father, the NIPT would indicate either a fetus affected with β-thalassaemia or a non-affected β-thalassaemia carrier (Table S8). This indicates the requirement for further investigation with invasive sampling procedures to determine the maternal inheritance and the final diagnosis. Nevertheless, the NIPH was successful for all families with one or more informative variants, while 8 out of the 17 families examined avoided further invasive procedures (47% of families).

## 3. Discussion

Early NIPT studies on single-gene disorders were focused on the analysis of the paternally inherited pathogenic variants in autosomal dominant disorders, as well as on autosomal recessive disorders for couples with different pathogenic variants. The reason behind this was the ease of the detection or exclusion of genetic sequences absent from the maternal genome by qualitative analyses. However, this approach is highly prone to misdiagnosis, as it is based on single-point analysis. The innovation of this study lies in the combinatory targeted NGS analysis of a large number of polymorphic and pathogenic SNVs, thus providing higher diagnostic accuracy and reliability. In addition, the analysis of multiple polymorphic SNVs allows indirect NIPT diagnosis, even for couples that share the same pathogenic variant. The ultimate goal of this study lies in a growing demand for the clinical implementation of a risk-free prenatal diagnostic method that can be universally applied, providing high sensitivity, accuracy, and specificity.

Taking advantage of the high analytical power that the NGS technology holds, this study has achieved the correct detection of the paternally inherited SNVs in cases of maternal homozygosity, demonstrating the extreme robustness, accuracy, and specificity of the method. In our first NGS-NIPT study [13], the analysis of 10 cfDNA samples for 4 informative SNVs was performed, for which the selective capture of β-globin gene cluster regions of interest was performed with conventional PCR before sequencing. The results obtained enabled the haplotype analysis of the families and in turn NIPT of 8 out of 10 families. Although the results were encouraging, the study highlighted a number of points that needed to be addressed in further optimization to improve detection efficiency. These points were, amongst others, the inclusion of a higher number of target variants, the design of shorter amplicons, and a higher concentration of cfDNA material for library preparation. 

Therefore, the present study aimed to improve the diagnostic efficiency of the previous work, taking into consideration the points highlighted. This was performed by broadening the panel of SNVs to 154 as opposed to 4, reducing amplicon sizes (40 bp–154 bp vs. 170 bp–268 bp) and increasing the amount of input cfDNA by using 2 mL instead of 1 mL of maternal plasma for cfDNA extraction. The aim was to eliminate or altogether abolish incidences of erroneous results inherent to the challenging cfDNA material. In addition, the amplification of all targets throughout the β-globin cluster was performed using a highly multiplexed primer panel specifically designed to improve amplification efficiency and at the same time reduce time and labor requirements. 

In order to achieve this, an in silico analysis initially identified all SNVs located on the β-globin gene cluster that exhibited MAF > 1% across Europe (Figure 2A). Subsequently, a targeted NGS assay was performed for the determination of the highly heterozygous SNVs, resulting in 219 variants. These SNVs were targeted together with the entire HBB gene for inclusion in a custom genotyping panel for cfDNA-NGS analysis. The optimization of the final design was achieved while taking into consideration a number of parameters by using special design tools to ensure the maximum possible capture of fetal fragments in a highly multiplexed manner. The final panel consists of 157 amplicons that cover almost the entire HBB gene and 154 highly polymorphic SNVs across the β-globin gene cluster (Figure 2A).

The importance of targeting and analyzing a large number of SNVs is significant as it overcomes the need for bespoke test development, offering wide applicability and uniformity. Importantly, it can be beneficial for couples who share the same pathogenic variant, and at the same time it increases the number of informative variants that can be interrogated per family during NGS. As opposed to single-point detection approaches, the analysis of multiple polymorphisms offers increased diagnostic accuracy and specificity, which are of conceptual importance in prenatal diagnostics and especially in NIPT, where the fetal genome is usually masked by the large number of maternal sequences within maternal plasma.

The determination of the paternally transmitted haplotype can be beneficial to 50% of the cases where the fetus inherits the wild-type paternal allele (Figure 2B). In this scenario, the fetus is either a carrier, where the β-thalassaemia maternal allele is inherited, or the β-thalassaemia allele is absent, where the wild-type maternal allele is transmitted to the fetus. In both cases, the fetus is unaffected; therefore, invasive procedures are avoided. Instead, for inheritance of the β-thalassaemia paternal allele, the fetus could be homozygous-affected if the β-thalassaemia allele is also inherited from the mother, or could be an unaffected carrier if the wild-type maternal allele is transmitted. It would then be necessary to determine the carrier status of the fetus via further procedures.

The efficiency of our custom, high-density HAPLONID panel was assessed on 17 cfDNA samples of at-risk pregnancies. The approach used to determine the cutoff value for fetal allele detection was empirical and was based on adding one SD to the maximum error rate observed per family. This method provided reliable results and improved detection rates compared to alternative thresholds. However, further work is needed to refine and calibrate the cutoff value, potentially incorporating larger datasets or alternative, more sophisticated statistical methodologies to enhance its precision and applicability. The true positive and true negative detection of the paternally inherited fetal alleles was achieved in 453 out of the 481 variants analyzed, with only minimal discordance in families 1, 5, 10, 11, 15, and 17. Families 2, 3, and 4 showed consistent results between the two cfDNA replicates. In contrast, family 1 presented variation between the two (7/11 as opposed to 9/11 true positive detection), thus indicating the great importance of having cfDNA replicates in NIPT assays. Although we did not have full concordance in families 1, 5, 10, 11, 15, and 17, the inclusion of a high number of SNVs increased the possibility of having concordant SNVs, therefore allowing us to safely link the fetal allele with the paternal allele. As the number of informative variants in these families was high and the true positive and true negative allele detection was achieved in more than 50% of those, our analysis was directed towards diagnostic interpretation.

It is crucial to highlight that, despite the minimal occurrence of false negatives and false positives observed in these six families, successful linkage with the correct paternal haplotype was achieved. The importance of having a larger number of SNVs to analyzed for an increased accuracy of diagnosis is demonstrated within the analysis of these families. To establish a cfDNA–paternal haplotype concordance threshold, below which an inconclusive result would be inferred and the repetition of the assay would be requested, further analysis of additional families is necessary.

False-negative SNV calls could be attributed to the random fragmentation of fetal DNA, which can prevent the primer from annealing at specific locations. In view of this limitation, one could use multiple overlapping primer pairs for each targeted SNV instead of one, as is proposed by Xiong et al. [32]. In addition, since the fetal fraction is the key point of the whole NIPT concept, our efforts should also concentrate on increasing the fetal fraction obtained. Therefore, as proposed by different groups [28,33], the collection of maternal peripheral blood could be performed in specific tubes containing preservatives, like the Cell-free DNA BCT^®^ tubes (Streck, La Vista, NE, USA), to prevent the gDNA release from maternal nucleated blood cells, thus enhancing the increased extraction of cfDNA. Furthermore, other groups have also demonstrated that the utilization of more than 2 mL of maternal plasma for cfDNA extraction would increase the concentration of the extracted cfDNA, thus increasing the initial amount added in the amplification reaction [29,34,35].

False-positive SNV calls could be attributed to some erroneous alignments of short reads that might have been caused by the high number of cycles used in PCR, thus also amplifying nonspecific fragments. This can be overcome after the optimization of the PCR cycling program by testing a range of PCR cycles. 

In an effort to assess the efficiency and the feasibility of our custom NGS-SNV panel in order to use it in the NIPT of β-thalassaemia, the construction of haplotypes was performed for paternal inheritance inference in 16 out of 17 families examined, for which at least one informative SNV was identified. Of those, three also included samples from the grandparents (i.e., families 2, 16 and 17), demonstrating a real case scenario. In the case where the grandparental samples are not available, one can perform direct chromosomal phasing using droplet digital PCR [36] or long-read sequencing [37] for the construction of haplotypes and the linkage of the inferred haplotypes with the disease under study, enabling the feasibility of the proposed NIPT. 

In this study, we presented that non-invasive prenatal haplotyping was effectively achieved for 16 out of 17 cfDNA samples examined, for which at least one informative SNV was identified, demonstrating correct diagnostic interpretation after cfDNA analysis. The paternally inherited fetal haplotype was correctly determined in the cfDNA with 100% concordance with the CVS haplotype in 10 out of the 16 families examined (i.e., families 2, 3, 4, 6, 7, 8, 9, 12, 13, and 16). Importantly, the correct determination of the paternal inheritance was also accomplished for families 1 (cfDNA sample ID: 275_a and 275_b), 5, 15, and 17, where the true positive detection of the paternal allele was observed in 7/11 (64%), 9/11 (82%), 51/67 (76%), 29/30 (97%), and 24/26 (92%) of the informative SNVs, respectively. Similarly, the paternal inheritance was also successfully determined for families 10 and 11, where the true negative detection of the paternal allele was observed in 5/6 (83%) and 68/70 (97%) of the informative SNVs, respectively. This highlights the high diagnostic accuracy of our NIPT approach, which analyzes a large number of polymorphisms. This ensures the correct interpretation of fetal inheritance, even in cases of discordant results, by comparing the inferred fetal haplotypes with the parental haplotypes. An advantage of the suggested haplotype approach is its ability to identify discordant SNVs, which leads to inconclusive results and thereby prevents misdiagnosis. In 4 out of the 16 families for which NIPH was performed (i.e., families 3, 7, 9 and 16), the correct detection of the inherited paternal haplotype was based on the true negative detection of the minor paternal allele, confirmed with the CVS haplotypes. Nonetheless, in a real-case scenario where the CVS sample is unavailable, the distinction between fetal and maternal haplotypes must be unambiguous. As the possibility of detecting very similar haplotypes between the parents is high in Cyprus and other inbred populations, the introduction of more highly heterozygous SNVs and of a fetal DNA-specific marker in our custom genotyping panel would overcome this limitation, providing evidence for a fetal DNA presence in the cfDNA sample. Furthermore, this will also provide NIPT applicability in consanguineous families. 

In conclusion, in 8 families where the fetus inherited the wild-type paternal allele, the need to perform invasive prenatal diagnosis was eliminated. Nonetheless, in 8 other cases, where the β-thalassaemia paternal allele was inherited, further investigation was necessary to determine maternal inheritance and finalize the prenatal diagnosis. 

This study proved the efficiency and robustness of our custom NGS genotyping panel in providing accurate NIPT of β-thalassaemia, demonstrating 98.8% specificity and 88.8% sensitivity for paternally inherited fetal allele detection and 100% success in final diagnostic interpretation. The successful detection of paternally inherited haplotype was accomplished in 16 out of 17 cfDNA samples examined, for which at least 1 informative SNV was identified, thus providing evidence for NIPT feasibility in 50% of the cases using targeted NGS and moving NIPT one step closer to the clinic. Subsequently, this assay can be combined with our group’s recently developed NIPT assay for the determination of the fetal genotype based on the most common β-thalassaemia pathogenic variant NM_000518.5:c.[93-21G>A] [14], strengthening the diagnostic power of the approach. The two outcomes of our analyses work synergistically for the detection of both paternally and maternally inherited fetal alleles, leading towards a final diagnosis, and thus covering all cases at risk. The proposed approach will soon be validated by performing a large-scale cfDNA analysis in parallel with the CVS analysis in order to render the assays ready to be implemented in the clinical setting. Moreover, future work includes the optimization of an NGS-based relative haplotype dosage (RHD) approach described by others [29,30,34,35,38], together with the addition of Unique Molecular Identifiers (UMIs) [39] in our custom targeted NGS panel, to provide the sensitive determination of the fetal genotype regardless of the familial pathogenic variant, thus completely replacing the invasive procedures. 

## 4. Materials and Methods

### 4.1. Sample Collection and DNA Extraction

Peripheral blood samples were collected from 96 random Greek–Cypriot individuals for the determination of the high-heterozygosity SNVs in the Cypriot population, located on the β-globin gene cluster. The custom, high-density NGS oligo panel was assessed for 17 families. Peripheral blood from the 17 couples at risk for β-thalassaemia in their fetus was collected, as well as maternal plasma samples from the pregnant women around the 10th week of gestation. All blood samples were collected into EDTA-containing tubes. Within 4–6 h of collection, the plasma was isolated from the cells by centrifugation at a low speed of 2500× *g* for 10 min without braking. It was then transferred to microcentrifuge tubes and subjected to a second centrifugation at 16,000× *g* for 40 min to eliminate any remaining cells [40]. The plasma samples were subsequently stored at −20 °C until extraction. Genomic DNA samples were extracted from whole blood using the Puregene Blood Core Kit C according to the manufacturer’s instructions (Qiagen Sciences, Germantown, MD, USA). Cell-free DNA was extracted from 2 mL of maternal plasma using QIAamp Circulating Nucleic Acid Kit (Qiagen GmbH, Hilden, Germany) and eluted into 65 μL (families 1–9) and 25 μL (families 10–17) of AVE buffer, following the manufacturer’s protocol. 

### 4.2. SNV Selection

In order to identify the most highly heterozygous variants in our population and increase the possibility of having a high number of informative SNVs for each family, all 307 SNVs located on the β-globin cluster were retrieved from the Ensembl BioMart, and we set a minor allele frequency (MAF) of more than 1% in Europe as a filter (Figure 2A). 

### 4.3. Long-Range PCR 

Then, 8 sets of primers (Table 5) were designed to target the pre-identified 307 SNVs, yielding amplicons of approximately 10 Kb each to amplify the whole β-globin gene cluster and identify the high-heterozygosity SNVs in our population. The Vector NTI 11.5.4 software was used to design the primers using the reference sequence of the hemoglobin gene locus based on human genome assembly GRCh38 with accession number NG_000007, which was downloaded from the NCBI database. 

The generation of specific PCR products with large sizes is challenging; therefore, the optimization process entailed a repetitive series of trials. Based on these trials, a touchdown long-range PCR protocol was found to be the best option for achieving the required specificity. We used 35 ng of gDNA as a template in a reaction volume of 20 μL that contained 4 μL of 5X PrimeSTAR GXL buffer (Mg^2+^ plus), 0.4 μL of DMSO, 1.6 μL of 2.5 mM dNTP mixture, 1.2 μL of each 2.5 μM primer, and 0.4 μL of 1.25 U/μL PrimeSTAR GXL DNA polymerase (Clontech Laboratories, Inc., A Takara Bio Company, Mountain View, CA, USA).

The PCR conditions were as follows. The first part entailed 98 °C treatment for three min; there were 12 cycles at 98 °C for 10 s and at 70 °C for 30 s in the first cycle, with a reduction of 1 °C per cycle and extension at 68 °C for 9 min. The second part entailed 20 cycles at 98 °C for 10 s, 20 cycles at 62 °C for 30 s, 20 cycles at 68 °C for 9 min, and a final extension at 68 °C for 10 min.

All reactions were performed using the Applied Biosystems Veriti 96 Fast Thermal Cycler (Applied Biosystems, Foster City, CA, USA) and the specificity of the amplicons of interest was confirmed after agarose gel electrophoresis.

### 4.4. Next-Generation Sequencing of Long-Range PCR Amplicons

The long-range PCR products were purified using the MinElute 96 UF PCR Purification Kit (Qiagen, Hilden, Germany) according to the manufacturer’s instructions. The PCR products were then assessed for impurities using the NanoDrop 2000 UV–Vis spectrophotometer (ThermoFisher Scientific, Waltham, MA, USA), measured on a Qubit fluorometer using the Qubit dsDNA HS Kit (ThermoFisher Scientific, Waltham, MA, USA), and diluted down to 1 ng each.

Then, we pooled together 3 μL of each of the 8 amplicons (1 ng/μL) per sample, obtaining a total concentration of 24 ng, and proceeded to the library preparation as per the Illumina DNA-prep protocol’s instructions. The final pool, consisting of 96 libraries, was loaded onto the MiSeq Reagent Kit v2 (300 cycles) and sequenced on a MiSeq Illumina system at a final concentration of 12 pM, as per the protocol’s recommendations.

### 4.5. Downstream Analysis of Illumina DNA Prep Libraries

First, raw data bcl2 files were converted and demultiplexed into FASTQ using bcl2fastq2 conversion software (v2.20). Subsequently, adaptor sequences were trimmed using the Adaptor trimmer (v 1.0) (https://github.com/erasmus-center-for-biomics/AdapterTrimmer/) (URL accessed on 29 March 2018) to remove adaptor sequences and prevent potential alignment errors. Next, via the BWA MEM software (v 0.7.12) [41], the sequencing reads were aligned with the human reference genome assembly GRCh38 and stored as BAM files. The alignments were further processed with Picard v2.7.11 and variants were determined with the Genome Analysis Toolkit (GATK) software v4.0.2.0. 

### 4.6. Design of the High-Density Custom Genotyping Panel Haplonid

A custom, high-density Ampliseq (Illumina, San Diego, CA, USA) panel that targets the pre-identified, highly heterozygous SNVs and the HBB gene was designed (Figure 2A), fulfilling the following requirements for cfDNA analysis. The primers included in the design should not overlap with common variants in our population as this would likely lead to PCR bias and amplicon dropout in specific individuals. The designed amplicons should be between 100 and 125 bp (with an absolute maximum of 140 bp) to maximize yield. Since the prominence size of cffDNA is ~143 bp [29], small amplicons would increase the chances of capturing variations. Importantly, the 15 most common β-thalassaemia pathogenic SNVs in Cyprus should be covered and included in the oligonucleotide panel [25], enabling the genotyping of the parents and direct paternal inheritance determination in cfDNA in case parents share different pathogenic variants. Also, as many of the pre-determined, highly heterozygous SNVs need to be covered as possible in order to increase the chance of having enough informative SNVs for each family to elucidate the fetal haplotypes reliably. Finally, as much of the HBB gene region as possible should be covered to identify secondary genetic variation. 

The custom oligo panel is split into two primer pools, allowing the creation of overlapping amplicons for HBB gene coverage. Primer pool one consists of 79 primer pairs and pool two consists of 78 primer pairs.

### 4.7. Sample Selection for the High-Density Custom-Designed Genotyping Panel

In an effort to evaluate the specificity and sensitivity of the custom primer pools, an Ampliseq for Illumina assay (San Diego, CA, USA) was performed. For this study, 17 families of Greek–Cypriot origin with known mutational statuses and at risk of having a β-thalassaemia-affected fetus were selected for analysis with the AmpliSeq for Illumina assay (San Diego, CA, USA) (Figure 2B) (Table 6). Fourteen of the seventeen families included the gDNA samples of the couple and the cfDNA sample extracted from maternal plasma. The corresponding CVS sample of each pregnancy was also included in the experiment for direct and parallel comparison of the results obtained from the cfDNA samples, as well as for wild-type/β-thalassaemia haplotype determination. In routine NIPT application, the sequencing of the grandparents of each family was conducted where possible to determine the phase of the parental haplotypes. For the proof of concept, the grandparents in families 2, 16, and 17 were also included, demonstrating a real case clinical scenario. Hence, the analysis of 63 gDNA samples and 17 cfDNA samples was performed. For comparison reasons, it was decided to use two replicate reactions for each cfDNA sample of families 1, 2, 3, and 4 and one cfDNA reaction for families 5–17. Moreover, to simulate the fetomaternal characteristics of cfDNA and create samples of known ‘fetal fraction’, artificial gDNA spikes were generated and analyzed in parallel with the 17 families, resulting in 88 reactions in total. The spiked samples were created by diluting the gDNA samples of the couple in family 5 down to 0.2 ng/μL and spiking the paternal DNA (53213) to the maternal DNA (53212) in ratios of 1.25%, 2.5%, 5%, and 10%.

### 4.8. Ampliseq for Illumina Library Preparation and Sequencing

Genomic DNA samples were quantified using the Quant-iT dsDNA Broad-Range Assay Kit for families 1–9 and the Qubit dsDNA Broad-Range Assay Kit for families 10–17. Moreover, the determination of the cfDNA sample concentration was performed using the Quant-iT dsDNA High-Sensitivity Assay Kit for families 1–9 and the Qubit dsDNA High-Sensitivity Assay Kit for families 10–17. Then, 10 nanograms of the gDNA samples and between 0.525 and 12.8 ng of the cfDNA samples were used as inputs and amplified for 19 and 22 cycles, respectively, following the two-primer pool AmpliSeq for Illumina library preparation. The prepared libraries were assessed in terms of quantity and quality. We used the Agilent 2100 Bioanalyzer (Agilent Technologies, Santa Clara, CA, USA) and the Agilent DNA 1000 Kit (Agilent Technologies, Santa Clara, CA, USA) for families 1–9 and the Agilent Tapestation 2200 (Agilent Technologies, Santa Clara, CA, USA) and the high-sensitivity D1000 kit (Agilent Technologies, Santa Clara, CA, USA) for families 10–17.

Approximately 10K sequencing coverage was set as a goal based on the fact that the fetal DNA represents only a small percentage of the cfDNA [2,3], with great homology to the overwhelmed maternal genome. Therefore, a high number of reads representing the fetal genome are needed to reliably deduce the fetal genotype. The final libraries were split into 6 groups of 12 and 1 group of 16, each loaded onto a MiSeq Reagent Kit v3 (600-cycles), and we sequenced 150 paired-end cycles on a MiSeq Illumina system.

### 4.9. Downstream Analysis of Ampliseq for Illumina Libraries 

The relevant Illumina adapter sequence (CTGTCTCTTATA) was trimmed from the reads using the AdapterTrimmer tool (v 1.0). The reads were subsequently aligned to the human GRCh38 reference genome using BWA mem (v 0.7.12) [41]. Format conversions were performed using the SAMtools software (v 1.3.1) [42]. From the alignments, variants were established for all samples combined using BCFtools (v 1.3) [43]. For variant determination, a mapping quality threshold of 10 was set, probabilistic realignments were disabled, and anomalous read pairs were included. Variants were finally determined using the multiallelic caller included in BCFtools. The igvtools software (v 2.3) [44,45] was used for data extraction from BAM files in order to calculate the allele percentages of the gDNA samples for each analyzed variant. A custom, in-house Perl script was developed for the calculation of the error rate and the fetal fraction (*ff*) in the 21 cfDNA reactions and the error rate and the minor genotype fraction in the 4 gDNA-spiked samples, using information extracted from the VCF files. The calculations were based on the following equation [29]:

(1) $$ff(\%)=\frac{2p}{p+q}\times 100$$

This is the equation for fetal fraction (*ff)* calculation, where p is the read count of the fetal-specific allele and q is the number of the sequenced reads of the other allele, which is shared by the mother and the fetus.

### 4.10. Statistical Analysis

Statistical analysis was performed using the R programming language (version 4.2.2). In particular, the epiR package (2.0.52) was used for the calculation of sensitivity, specificity, and confidence intervals. More specifically, the epi.tests function with the clopper-pearson method was utilized for the calculation of 95% confidence intervals (95% CI).

## Figures and Tables

**Table 1:** Statistics for the fraction of the minor genotype within genomic DNA-spiked samples.

Sample Name	Informative SNVs	Mean Value (%)	SD (%)	Median Value (%)	Mean −1SD (%)	Mean +1SD (%)
Spike 1.25%	67	0.85	0.96	0.28	−0.12	1.81
Spike 2.5%	67	1.97	1.85	1.92	0.13	3.82
Spike 5%	67	2.95	1.63	2.58	1.32	4.58
Spike 10%	67	8.55	2.87	8.25	5.67	11.42

SD: standard deviation.

**Table 2:** The cut-off values for paternally inherited fetal allele detection.

Family ID	cfDNA ID	Max_ Error Rate	SD_ Error Rate	Cut-Off (MAX+1SD)
1	275_a	1.404	0.237	1.641
1	275_b	1.69	0.196	1.886
2	311_a	0.919	0.194	1.113
2	311_b	0.533	0.118	0.651
3	322_a	0.463	0.116	0.579
3	322_b	0.533	0.138	0.671
4	323_a	0.688	0.129	0.817
4	323_b	0.737	0.165	0.902
5	306	1.299	0.223	1.522
6	303	0.619	0.122	0.741
7	296	1.434	0.243	1.677
8	274	1.655	0.33	1.985
9	273	0.465	0.118	0.583
10	350	0.228	0.063	0.291
11	352	0.302	0.073	0.376
12	253	0.870	0.156	1.027
13	337	0.462	0.090	0.553
14	360	0.400	0.099	0.499
15	361	0.736	0.230	0.967
16	371	0.843	0.155	0.998
17	374	0.932	0.214	1.147

SD: standard deviation.

**Table 3:** Number of informative SNVs for each family.

Family ID	Number of Informative SNVs
1	11
2	3
3	22
4	8
5	67
6	10
7	70
8	5
9	27
10	19
11	71
12	4
13	61
14	0
15	31
16	1
17	27

**Table 4:** True and false detection rates obtained in this study.

Family ID	cfDNA ID	True Positive	False Negative	True Negative	False Positive
**1**	275_a	7/11 [64%]	4/11 [36%]	0/0	0/0
**1**	275_b	9/11 [82%]	2/11 [18%]	0/0	0/0
**2**	311_a	1/1 [100%]	0/1 [0%]	2/2 [100%]	0/2 [0%]
**2**	311_b	1/1 [100%]	0/1 [0%]	2/2 [100%]	0/2 [0%]
**3**	322_a	0/0	0/0	22/22 [100%]	0/22 [0%]
**3**	322_b	0/0	0/0	22/22 [100%]	0/22 [0%]
**4**	323_a	8/8 [100%]	0/8 [0%]	0/0	0/0
**4**	323_b	8/8 [100%]	0/8 [0%]	0/0	0/0
**5**	306	51/67 [76%]	16/67 [24%]	0/0	0/0
**6**	303	10/10 [100%]	0/10 [0%]	0/0	0/0
**7**	296	0/0	0/0	70/70 [100%]	0/70 [0%]
**8**	274	1/1 [100%]	0/1 [0%]	4/4 [100%]	0/4 [0%]
**9**	273	0/0	0/0	27/27 [100%]	0/27 [0%]
**10**	350	13/13 [100%]	0/13 [0%]	5/6 [83%]	1/6 [17%]
**11**	352	1/1 [100%]	0/1 [0%]	68/70 [97%]	2/70 [3%]
**12**	253	4/4 [100%]	0/4 [0%]	0/0	0/0
**13**	337	31/31 [100%]	0/31 [0%]	30/30 [100%]	0/30 [0%]
**14**	360	0/0	0/0	0/0	0/0
**15**	361	29/30 [97%]	1/30 [3%]	1/1 [100%]	0/1 [0%]
**16**	371	0/0	0/0	1/1 [100%]	0/1 [0%]
**17**	374	24/26 [92%]	2/26 [8%]	1/1 [100%]	0/1 [0%]

**Table 5:** Primer sequences and fragment sizes.

Amplicon	Primer F (5’-3’)	Primer R (5’-3’)	Amplicon Size (bp)
1	GCTAGTGTCTTAAGAGGTTCACATTT	CTCTAAGAAAGTTACAACATGGTGAAT	8760
2	GGACCTGGGAGGAGGGTTAT	CGTATGTGAGCATGTGTCCTCTAA	8362
3	GCAGAGCCTTGATGGGATTA	CCACTCAGGTCCTAACTCTAACTTT	9328
4	GATGATTCAAGGGGACAGATACAA	GAATGACCTGTTTATCACAACTGTT	9723
5	GGTTCACCTCAGTCTCTATAATCTGTA	GGCTAGAGTAAAGCATGTTGAAGTAA	9434
6	GGCACCATTAGCCAGAGAAT	GGCATGGTTTGATTTGTGTCTT	9615
7	CCAACAGAGTTTGAGTTTCTATTGAT	CCAAATAGTAATGTACTAGGCAGACTGT	9498
8	CATCAGTGTGGAAGTCTCAGGAT	CTACGGATGTGTGAGATCAGTTT	10,486

**Table 6:** Samples analyzed by the Ampliseq™ for Illumina^®^ assay.

Family	Sample ID	Source	Sample	Family Member	Genotype
**1**	50289	WBC	gDNA	Mother	NM_000518.5:c.[92+1G>A];[92+1=]
	50290	WBC	gDNA	Father	NM_000518.5:c.[316-106C>G];[316-106=]
	3871/50353	CVS	gDNA	Fetus	NM_000518.5:c.[92+1G>A]; [316-106C>G]
	275_a	MP	cfDNA	Mother and fetus	NA
	275_b	MP	cfDNA	Mother and fetus	NA
**2**	50321	WBC	gDNA	Mother	NM_000518.5:c.[93-21G>A];[93-21=]
	50322	WBC	gDNA	Father	NM_000518.5:c.[93-21G>A];[93-21=]
	4123/53660	CVS	gDNA	Fetus	NM_000518.5:c.[=];[=]
	311_a	MP	cfDNA	Mother and fetus	NA
	311_b	MP	cfDNA	Mother and fetus	NA
	50323	WBC	gDNA	Mother of mother	NM_000518.5:c.[93-21G>A];[93-21=]
	50324	WBC	gDNA	Father of mother	NM_000518.5:c.[=];[=]
	50325	WBC	gDNA	Mother of father	NM_000518.5:c.[=];[=]
	50326	WBC	gDNA	Father of father	NM_000518.5:c.[93-21G>A];[93-21=]
**3**	52205	WBC	gDNA	Mother	NM_000518.5:c.[93-21G>A];[93-21=]
	52206	WBC	gDNA	Father	NM_000518.5:c.[93-21G>A];[93-21=]
	4142/53887	CVS	gDNA	Fetus	NM_000518.5:c.[93-21G>A];[93-21 G>A]
	322_a	MP	cfDNA	Mother and fetus	NA
	322_b	MP	cfDNA	Mother and fetus	NA
**4**	53973	WBC	gDNA	Mother	NM_000518.5:c.[93-21G>A];[93-21=]
	41110	WBC	gDNA	Father	NM_000518.5:c.[93-21G>A];[93-21=]
	4147/53929	CVS	gDNA	Fetus	NM_000518.5:c.[93-21G>A];[93-21=]
	323_a	MP	cfDNA	Mother and fetus	NA
	323_b	MP	cfDNA	Mother and fetus	NA
**5**	53212	WBC	gDNA	Mother	NM_000518.5:c.[93-21G>A];[93-21=]
	53213	WBC	gDNA	Father	NM_000518.5:c.[93-21G>A];[93-21=]
	4100/53323	CVS	gDNA	Fetus	NM_000518.5:c.[93-21G>A];[93-21=]
	306	MP	cfDNA	Mother and fetus	NA
**6**	53195	WBC	gDNA	Mother	NM_000518.5:c.[118C>T];[118=]
	53196	WBC	gDNA	Father	NM_000518.5:c.[118C>T];[118=]
	4095/53252	CVS	gDNA	Fetus	NM_000518.5:c.[118C>T];[118=]
	303	MP	cfDNA	Mother and fetus	NA
**7**	52952	WBC	gDNA	Mother	NM_000518.5:c.[93-21G>A];[93-21=]
	52953	WBC	gDNA	Father	NM_000518.5:c.[93-21G>A];[93-21=]
	4081/52990	CVS	gDNA	Fetus	NM_000518.5:c.[93-21G>A];[93-21=]
	296	MP	cfDNA	Mother and fetus	NA
**8**	50040	WBC	gDNA	Mother	NM_000518.5:c.[92+1G>A];[92+1=]
	50041	WBC	gDNA	Father	NM_000518.5:c.[93-21G>A];[93-21=]
	3854/50140	CVS	gDNA	Fetus	NM_000518.5:c.[=];[=]
	274	MP	cfDNA	Mother and fetus	NA
**9**	49899	WBC	gDNA	Mother	NM_000518.5:c.[93-21G>A];[93-21=]
	49900	WBC	gDNA	Father	NM_000518.5:c.[93-21G>A];[93-21=]
	3836/49924	CVS	gDNA	Fetus	NM_000518.5:c.[93-21G>A];[93-21=]
	273	MP	cfDNA	Mother and fetus	NA
**10**	57203	WBC	gDNA	Mother	NM_000518.5:c.[93-21G>A];[93-21=]
	57204	WBC	gDNA	Father	NM_000518.5:c.[92+1G>A];[92+1=]
	57219/4359	CVS	gDNA	Fetus	NM_000518.5:c.[92+1G>A];[92+1=]
	350	MP	cfDNA	Mother and fetus	NA
**11**	57262	WBC	gDNA	Mother	NM_000518.5:c.[93-21G>A];[93-21=]
	57263	WBC	gDNA	Father	NM_000518.5:c.[92+1G>A];[92+1=]
	57261/4369	CVS	gDNA	Fetus	NM_000518.5:c.[92+1G>A];[92+1=]
	352	MP	cfDNA	Mother and fetus	NA
**12**	49166	WBC	gDNA	Mother	NM_000518.5:c.[93-21G>A];[93-21=]
	49167	WBC	gDNA	Father	NM_000518.5:c.[93-21G>A];[93-21=]
	49283/3789	CVS	gDNA	Fetus	NM_000518.5:c.[93-21G>A];[93-21=]
	253	MP	cfDNA	Mother and fetus	NA
**13**	56423	WBC	gDNA	Mother	NM_000518.5:c.[93-21G>A];[93-21=]
	12506	WBC	gDNA	Father	NM_000518.5:c.[92+6T>C];[-137C>G]
	56389/4301	CVS	gDNA	Fetus	NM_000518.5:c.[93-21G>A];[ 92+6T>C]
	337	MP	cfDNA	Mother and fetus	NA
**14**	50542	WBC	gDNA	Mother	NM_000518.5:c.[93-21G>A];[93-21=]
	50543	WBC	gDNA	Father	NM_000518.5:c.[93-21G>A];[93-21=]
	58209/4419	CVS	gDNA	Fetus	NM_000518.5:c.[93-21G>A];[93-21=]
	360	MP	cfDNA	Mother and fetus	NA
**15**	51468	WBC	gDNA	Mother	NM_000518.5:c.[118C>T];[118=]
	51469	WBC	gDNA	Father	NM_000518.5:c.[93-21G>A];[93-21=]
	58210/4420	CVS	gDNA	Fetus	NM_000518.5:c.[118C>T];[118=]
	361	MP	cfDNA	Mother and fetus	NA
**16**	59244	WBC	gDNA	Mother	NM_000518.5:c.[93-21G>A];[93-21=]
	59245	WBC	gDNA	Father	NM_000518.5:c.[93-21G>A];[93-21=]
	59283/4485	CVS	gDNA	Fetus	NM_000518.5:c.[93-21G>A];[93-21=]
	371	MP	cfDNA	Mother and fetus	NA
	59246	WBC	gDNA	Mother of mother	NM_000518.5:c.[=];[=]
	59247	WBC	gDNA	Father of mother	NM_000518.5:c.[93-21G>A];[93-21=]
	59248	WBC	gDNA	Mother of father	NM_000518.5:c.[93-21G>A];[93-21=]
	59249	WBC	gDNA	Father of father	NM_000518.5:c.[=];[=]
**17**	58552	WBC	gDNA	Mother	NM_000518.5:c.[93-21G>A];[93-21=]
	58553	WBC	gDNA	Father	NM_000518.5:c.[92+6T>C];[92+6=]
	59408/4492	CVS	gDNA	Fetus	NM_000518.5:c.[93-21G>A];[ 92+6T>C]
	374	MP	cfDNA	Mother and fetus	NA
	59344	WBC	gDNA	Mother of mother	NM_000518.5:c.[=];[=]
	59345	WBC	gDNA	Father of mother	NM_000518.5:c.[93-21G>A];[93-21=]
	59346	WBC	gDNA	Mother of father	NM_000518.5:c.[93-21G>A];[93-21=]
	59347	WBC	gDNA	Father of father	NM_000518.5:c.[92+6T>C];[92+6=]

WBC: white blood cells; CVS: chorionic villi sample; MP: maternal plasma; gDNA: genomic DNA; cfDNA: cell-free DNA; NA: not available.

## Data Availability

The original contributions presented in this study are included in the article/Supplementary Material. Further inquiries can be directed to the corresponding authors.

## Supplementary Materials

The following supporting information can be downloaded at: https://www.mdpi.com/article/10.3390/ijms26020570/s1.
