# Supplementary material for: Non-Invasive Determination of the Paternal Inheritance in Pregnancies at Risk for β-Thalassaemia by Analyzing Cell-Free Fetal DNA Using Targeted Next-Generation Sequencing
Source: Int J Mol Sci. 2025 Jan 10;26(2):570. doi: 10.3390/ijms26020570 (PMC11765003; doi:10.3390/ijms26020570)
Supplement: Supplementary file 1 [file ijms-26-00570-s001.zip › Supplementary.pdf]

Table S1. Two hundred nineteen SNVs showing high heterozygosity in the Cypriot population.

The variants included in our custom, high-density genotyping panel are highlighted with grey

Chr	*POS*	*rs number*	*REF*	*ALT*	*Total_ samples*	*g(1/1)*	*g(0/0)*	*g(0/1)*	*f(0/1)*
chr11	5228140	rs1003586	C	T	96	1	64	31	0.322917
chr11	5242347	rs10128555	C	G	96	70	1	25	0.260417
chr11	5242453	rs10128556	C	T	96	5	59	32	0.333333
chr11	5262932	rs10160305	T	C	96	7	60	29	0.302083
chr11	5221139	rs10160441	C	T	96	74	1	21	0.21875
chr11	5220738	rs10160615	T	C	96	76	1	19	0.197917
chr11	5261664	rs10160678	C	A	96	66	2	28	0.291667
chr11	5262949	rs10160747	C	T	96	7	60	29	0.302083
chr11	5261227	rs10160820	A	C	96	5	60	31	0.322917
chr11	5276800	rs10488675	A	G	96	13	43	40	0.416667
chr11	5247567	rs10488676	G	A	96	32	13	51	0.53125
chr11	5248354	rs1065686	T	A	96	66	2	28	0.291667
chr11	5260304	rs10734494	C	T	96	66	2	28	0.291667
chr11	5227411	rs10742583	G	A	96	77	1	18	0.1875
chr11	5294562	rs10742614	C	G	96	61	6	29	0.302083
chr11	5221955	rs10768679	T	C	96	77	1	18	0.1875
chr11	5224277	rs10768682	G	A	96	77	1	18	0.1875
chr11	5226561	rs10768683	C	G	96	78	1	17	0.177083

chr11	5240009	rs10768687	C	G	96	36	11	49	0.510417
chr11	5252692	rs10768707	T	C	96	66	2	28	0.291667
chr11	5265926	rs10768733	C	T	96	66	2	28	0.291667
chr11	5270642	rs10768737	T	C	96	34	14	48	0.5
chr11	5289803	rs10768774	G	A	95	62	5	28	0.294737
chr11	5294589	rs10768778	A	G	96	36	15	45	0.46875
chr11	5216516	rs10837598	G	C	96	77	1	18	0.1875
chr11	5219221	rs10837601	C	T	96	11	54	31	0.322917
chr11	5220268	rs10837604	G	A	96	11	54	31	0.322917
chr11	5221253	rs10837614	A	T	96	74	1	21	0.21875
chr11	5221273	rs10837615	A	G	96	74	1	21	0.21875
chr11	5222329	rs10837620	G	A	96	11	54	31	0.322917
chr11	5222383	rs10837621	C	T	96	11	54	31	0.322917
chr11	5223069	rs10837626	T	A	96	77	1	18	0.1875
chr11	5223174	rs10837628	A	G	96	11	54	31	0.322917
chr11	5224812	rs10837630	G	C	96	77	1	18	0.1875
chr11	5225126	rs10837631	T	A	96	10	54	32	0.333333
chr11	5236808	rs10837643	T	A	96	36	11	49	0.510417
chr11	5259788	rs10837697	G	C	96	7	60	29	0.302083
chr11	5265578	rs10837707	T	C	96	34	15	47	0.489583
chr11	5280814	rs10837757	A	G	96	2	84	10	0.104167
chr11	5296323	rs10837767	T	G	96	36	15	45	0.46875
chr11	5221383	rs11036336	T	C	96	74	1	21	0.21875
chr11	5221468	rs11036338	G	C	96	11	54	31	0.322917
chr11	5224770	rs11036351	C	T	96	40	18	38	0.395833
chr11	5227774	rs11036364	A	G	96	16	27	53	0.552083
chr11	5241552	rs11036415	A	C	96	70	1	25	0.260417

chr11	5244450	rs11036431	G	A	96	5	58	33	0.34375
chr11	5253948	rs11036474	T	C	96	5	58	33	0.34375
chr11	5254010	rs11036475	G	A	96	32	15	49	0.510417
chr11	5254113	rs11036476	C	T	96	32	15	49	0.510417
chr11	5258792	rs11036496	G	C	96	34	15	47	0.489583
chr11	5261547	rs11036503	G	A	96	36	15	45	0.46875
chr11	5261559	rs11036504	G	A	96	36	15	45	0.46875
chr11	5262292	rs11036506	A	G	96	NA	83	13	0.135417
chr11	5262916	rs11036507	C	T	96	34	15	47	0.489583
chr11	5272915	rs11036562	C	A	96	34	14	48	0.5
chr11	5287498	rs11036634	C	T	96	39	12	45	0.46875
chr11	5287666	rs11036635	G	A	96	5	62	29	0.302083
chr11	5289276	rs11036639	A	C	95	36	15	44	0.463158
chr11	5289348	rs11036641	A	G	96	3	62	31	0.322917
chr11	5289833	rs11036644	A	T	95	33	15	47	0.494737
chr11	5294637	rs11036657	C	T	96	20	29	47	0.489583
chr11	5264726	rs112857819	T	C	96	NA	84	12	0.125
chr11	5262780	rs113651675	T	C	96	5	60	31	0.322917
chr11	5220292	rs11500399	A	T	96	1	83	12	0.125
chr11	5218221	rs11511924	G	A	96	57	4	35	0.364583
chr11	5268076	rs11822578	G	C	96	66	2	28	0.291667
chr11	5232247	rs12222763	G	C	96	22	19	55	0.572917
chr11	5259051	rs12284340	C	T	96	4	63	29	0.302083
chr11	5216835	rs12284572	G	A	96	11	54	31	0.322917
chr11	5217021	rs12284650	G	A	96	11	54	31	0.322917
chr11	5223699	rs12290192	A	C	96	40	19	37	0.385417
chr11	5270506	rs12295181	C	T	96	NA	82	14	0.145833

chr11	5222914	rs12364872	A	G	96	40	19	37	0.385417
chr11	5243876	rs12417960	C	A	96	5	58	33	0.34375
chr11	5216405	rs12785330	G	A	96	NA	79	17	0.177083
chr11	5216421	rs12785345	G	C	96	NA	79	17	0.177083
chr11	5216163	rs12786358	C	G	96	NA	79	17	0.177083
chr11	5225365	rs12788013	C	G	96	NA	79	17	0.177083
chr11	5217502	rs139043490	G	A	96	13	45	38	0.395833
chr11	5218633	rs139797435	A	G	96	11	54	31	0.322917
chr11	5219439	rs141350785	G	C	96	1	83	12	0.125
chr11	5218907	rs141504397	A	G	96	NA	79	17	0.177083
chr11	5225911	rs1609812	G	A	96	78	1	17	0.177083
chr11	5288465	rs16912979	T	C	96	5	61	30	0.3125
chr11	5254572	rs1894398	G	A	96	32	15	49	0.510417
chr11	5255588	rs2011051	G	T	96	33	15	48	0.5
chr11	5249648	rs202216517	G	A	96	38	15	43	0.447917
chr11	5253487	rs2070972	A	C	96	33	15	48	0.5
chr11	5254177	rs2070973	T	C	96	32	15	49	0.510417
chr11	5242916	rs2071348	T	G	96	5	59	32	0.333333
chr11	5238497	rs2105819	G	C	96	36	11	49	0.510417
chr11	5248701	rs2187608	G	C	96	7	58	31	0.322917
chr11	5224176	rs2187610	G	C	96	42	18	36	0.375
chr11	5253037	rs2236794	C	T	96	33	15	48	0.5
chr11	5252311	rs2255519	G	A	96	33	15	48	0.5
chr11	5248569	rs28440105	A	C	96	66	2	28	0.291667
chr11	5251452	rs2855036	C	T	96	7	58	31	0.322917
chr11	5250924	rs2855038	T	C	96	32	15	49	0.510417
chr11	5250441	rs2855039	C	T	96	5	58	33	0.34375

chr11	5256061	rs2855121	C	T	96	5	59	32	0.333333
chr11	5256006	rs2855122	C	T	96	35	15	46	0.479167
chr11	5255848	rs2855123	A	T	96	33	15	48	0.5
chr11	5252457	rs2855125	T	G	96	33	15	48	0.5
chr11	5251917	rs2855126	C	G	96	32	15	49	0.510417
chr11	5248352	rs34752752	AG	A	96	66	2	28	0.291667
chr11	5253222	rs34879481	G	GT	96	7	59	30	0.3125
chr11	5294668	rs35423254	C	T	94	19	28	47	0.5
chr11	5249833	rs368698783	C	T	96	5	59	32	0.333333
chr11	5270962	rs3759067	C	T	96	67	2	27	0.28125
chr11	5270600	rs3759069	A	G	96	34	14	48	0.5
chr11	5270398	rs3759070	C	G	96	6	55	35	0.364583
chr11	5270302	rs3759071	G	A	96	34	14	48	0.5
chr11	5237035	rs3759073	T	C	96	36	11	49	0.510417
chr11	5236548	rs3759074	G	A	96	4	59	33	0.34375
chr11	5234682	rs3813727	A	G	96	22	19	55	0.572917
chr11	5297150	rs3888708	C	A	96	35	15	46	0.479167
chr11	5237260	rs4283007	A	T	96	38	11	47	0.489583
chr11	5236932	rs4320977	A	G	96	35	12	49	0.510417
chr11	5263748	rs4348933	A	G	96	34	15	47	0.489583
chr11	5237362	rs4402323	C	T	96	36	11	49	0.510417
chr11	5237199	rs4426158	T	C	96	36	11	49	0.510417
chr11	5291171	rs4601817	A	G	95	36	12	47	0.494737
chr11	5237597	rs4910543	G	C	96	36	11	49	0.510417
chr11	5237626	rs4910544	T	A	96	36	11	49	0.510417
chr11	5244245	rs4910545	C	T	96	66	1	29	0.302083
chr11	5265082	rs4910546	T	C	96	34	15	47	0.489583

chr11	5272890	rs4910547	G	C	96	66	2	28	0.291667
chr11	5273171	rs4910548	C	T	96	66	2	28	0.291667
chr11	5237622	rs4910735	G	A	96	36	11	49	0.510417
chr11	5237759	rs4910736	C	A	96	36	11	49	0.510417
chr11	5238059	rs4910737	C	T	96	35	11	50	0.520833
chr11	5238062	rs4910738	T	A	96	35	11	50	0.520833
chr11	5244136	rs4910739	G	C	96	68	1	27	0.28125
chr11	5266060	rs4910740	G	A	96	34	13	49	0.510417
chr11	5288920	rs4910743	T	C	95	36	14	45	0.473684
chr11	5257923	rs5010979	T	C	96	66	2	28	0.291667
chr11	5258097	rs5010980	C	G	96	66	2	28	0.291667
chr11	5258125	rs5010981	C	A	96	66	2	28	0.291667
chr11	5258258	rs5010984	T	C	96	66	2	28	0.291667
chr11	5218376	rs541299175	A	ATAAGCTTTTTGATGTGC TGCTGGAT	96	61	4	31	0.322917
chr11	5296292	rs56237826	T	TA	96	37	15	44	0.458333
chr11	5232232	rs5789380	TATAAAAA	T	96	22	19	55	0.572917
chr11	5248356	rs5789383	A	AG	96	66	2	28	0.291667
chr11	5290120	rs61893113	GTCTA	G	96	65	4	27	0.28125
chr11	5216054	rs6578587	A	G	96	51	11	34	0.354167
chr11	5231021	rs6578588	T	C	96	37	10	49	0.510417
chr11	5238304	rs6578590	T	C	96	68	1	27	0.28125
chr11	5245731	rs6578591	T	C	96	68	1	27	0.28125
chr11	5247910	rs6578592	C	A	96	66	2	28	0.291667
chr11	5272395	rs6578594	A	T	96	66	2	28	0.291667
chr11	5273948	rs6578595	A	G	96	65	3	28	0.291667
chr11	5274401	rs6578596	A	G	96	61	3	32	0.333333

chr11	5269140	rs67385638	C	G	96	7	59	30	0.3125
chr11	5225282	rs7110263	T	G	96	77	1	18	0.1875
chr11	5287848	rs7119142	A	G	96	5	61	30	0.3125
chr11	5262937	rs7121303	T	C	96	66	2	28	0.291667
chr11	5216268	rs7128069	C	G	96	51	11	34	0.354167
chr11	5274874	rs7130110	G	C	96	61	3	32	0.333333
chr11	5227013	rs713040	A	G	96	78	1	17	0.177083
chr11	5262817	rs7131677	G	A	96	66	2	28	0.291667
chr11	5237463	rs72869882	C	T	96	1	79	16	0.166667
chr11	5267909	rs72872548	C	A	96	7	61	28	0.291667
chr11	5268823	rs72872549	C	T	96	5	61	30	0.3125
chr11	5288939	rs72872555	G	A	95	3	61	31	0.326316
chr11	5293129	rs72872557	T	C	96	3	67	26	0.270833
chr11	5232356	rs74049332	G	A	96	1	83	12	0.125
chr11	5251565	rs74049345	G	A	96	NA	84	12	0.125
chr11	5227599	rs74234654	T	A	96	49	7	40	0.416667
chr11	5271883	rs7479652	T	C	96	67	2	27	0.28125
chr11	5247176	rs7480197	C	T	96	32	13	51	0.53125
chr11	5226503	rs7480526	A	C	96	40	18	38	0.395833
chr11	5261278	rs7480802	T	C	96	36	15	45	0.46875
chr11	5261976	rs7480910	A	G	96	36	15	45	0.46875
chr11	5266705	rs7481986	C	T	96	34	14	48	0.5
chr11	5238189	rs7482694	G	A	96	36	11	49	0.510417
chr11	5247392	rs7483789	T	G	96	32	13	51	0.53125
chr11	5218465	rs76167052	T	G	96	11	54	31	0.322917
chr11	5217934	rs76788240	T	A	96	11	54	31	0.322917
chr11	5253693	rs778725378	C	CGT	96	33	15	48	0.5

chr11	5224973	rs78928216	A	C	96	40	18	38	0.395833
chr11	5245498	rs7924684	C	T	96	33	13	50	0.520833
chr11	5293687	rs7928927	C	T	95	35	15	45	0.473684
chr11	5222527	rs7930833	G	C	96	77	1	18	0.1875
chr11	5295501	rs7933082	G	C	95	36	14	45	0.473684
chr11	5243699	rs7934275	A	G	96	70	1	25	0.260417
chr11	5228938	rs7936823	G	A	96	26	17	53	0.552083
chr11	5218413	rs7943713	A	G	95	75	1	19	0.2
chr11	5226496	rs7946748	G	A	96	NA	79	17	0.177083
chr11	5235201	rs7948416	A	C	96	68	1	27	0.28125
chr11	5235417	rs7948668	A	G	96	34	12	50	0.520833
chr11	5248113	rs916111	T	A	96	32	15	49	0.510417
chr11	5217378	rs9666998	G	T	96	2	65	29	0.302083
chr11	5239346	rs968856	T	C	96	35	12	49	0.510417
chr11	5239228	rs968857	T	C	96	35	12	49	0.510417
chr11	5217638	rs9704754	G	A	96	76	1	19	0.197917
chr11	5291631	rs977711420	T	A	96	NA	85	11	0.114583
chr11	5216895	rs12279992	T	C	96	11	54	31	0.322917
chr11	5217205	rs9704069	C	T	96	11	54	31	0.322917
chr11	5217276	rs4258407	A	G	96	11	54	31	0.322917
chr11	5218962	NA	GT	G	96	62	3	31	0.322917
chr11	5219107	rs149518650	C	T	96	1	83	12	0.125
chr11	5220405	rs10837605	C	G	96	11	54	31	0.322917
chr11	5251323	NA	G	GA	96	7	59	30	0.3125
chr11	5259609	NA	TA	T	96	5	60	31	0.322917
chr11	5267998	NA	A	AGTTTT	96	5	61	30	0.3125
chr11	5268666	NA	A	AAAAC	96	66	2	28	0.291667

chr11	5270333	NA	G	GT	96	34	14	48	0.5
chr11	5271572	rs886851205	CTG	C	96	34	14	48	0.5
chr11	5272002	NA	T	TA	96	17	33	46	0.479167
chr11	5272904	NA	AT	A	96	34	14	48	0.5
chr11	5276193	NA	ATATG	A	96	NA	78	18	0.1875
chr11	5276915	NA	AG	A	96	2	83	11	0.114583
chr11	5242068	NA	ACATAGGTACAAACATAGTGGAC	A	96	NA	78	18	0.1875
chr11	5293284	NA	G	GGTTTCACT	96	2	68	26	0.270833
chr11	5250078	NA	C	CTGCT	96	66	2	28	0.291667
chr11	5248303	NA	GT	G	96	1	62	33	0.34375
chr11	5237873	NA	G	GAC	96	36	11	49	0.510417
chr11	5254268	rs33993529	T	G	96	NA	64	32	0.333333

Table S2. Eighty most common globally β-thalassaemia mutations covered by our assay.

Chr	*Common name*	*Start position (bp)*	*End position (bp)*	*HGVS name*
chr11	Poly_A_(A>G)_AATAAA>AATAGA	5225485	5225486	NM_000518.4:c.*112A>G
chr11	CD_127/128_-AGG_[Glu-Ala>Pro]	5225656	5225658	NM_000518.4:c.383_385delAGG
chr11	CD_127_(CAG>TAG)_Gln_to_Term_CD_(127aa)	5225659	5225660	NM_000518.4:c.382C>T
chr11	CD_124-126_(+CCA)_+Pro	5225662	5225663	NM_000518.4:c.378_379insCCA
chr11	CD_124_(-A)_>156aa	5225666	5225667	NM_000518.4:c.375delA
chr11	CD_121_GAA>TAA_(120aa)	5225677	5225678	NM_000518.4:c.364G>T
chr11	CD_121_GAA>CAA_[Glu>Gln]	5225677	5225678	NM_000518.4:c.364G>C
chr11	CD_114_(CTG>CCG)_Leu_to_Pro	5225697	5225698	NM_000518.4:c.344T>C
chr11	CD_110_CTG>CCG_[Leu>Pro]	5225709	5225710	NM_000518.4:c.332T>C
chr11	CD_106/107_(+G)	5225719	5225720	NM_000518.4:c.321_322insG
chr11	IVS_II-850_(-G)	5225726	5225727	NM_000518.4:c.316-1delG
chr11	IVS_II-848_(C>G)	5225728	5225729	NM_000518.4:c.316-3C>G
chr11	IVS_II-848_(C>A)	5225728	5225729	NM_000518.4:c.316-3C>A
chr11	IVS_II-745_C>G	5225831	5225832	NM_000518.4:c.316-106C>G
chr11	IVS_II-705_(T>G)	5225871	5225872	NM_000518.4:c.316-146T>G
chr11	IVS_II-654_C>T	5225922	5225923	NM_000518.4:c.316-197C>T
chr11	IVS_II-1_G>A	5226575	5226576	NM_000518.4:c.315+1G>A
chr11	CD_90_GAG>TAG	5226620	5226621	NM_000518.4:c.271G>T
chr11	CD_89/90_-GT	5226621	5226622	NM_000518.4:c.269_270delGT
chr11	CD_84-86_+T	5226632	5226633	NM_000518.4:c.258_259insT
chr11	CD_84/85_(+C)	5226635	5226636	NM_000518.4:c.255_256insC
chr11	CD_82/83_(-G)	5226641	5226642	NM_000518.4:c.250delG
chr11	CD_71/72_+A	5226674	5226675	NM_000518.4:c.216_217insA
chr11	CD_51_-C	5226737	5226738	NM_000518.4:c.154delC

chr11	CD_44_-C	5226756	5226757	NM_000518.4:c.135delC
chr11	CD_39_(CAG>TAG)	5226773	5226774	NM_000518.4:c.118C>T
chr11	CD_38/39_(-C)	5226775	5226776	NM_000518.4:c.116delC
chr11	CD_37_(TGG>TGA)	5226777	5226778	NM_000518.4:c.114G>A
chr11	CD_37_(TGG>TAG)	5226778	5226779	NM_000518.4:c.113G>A
chr11	CD_36/37_(-T)	5226779	5226780	NM_000518.4:c.112delT
chr11	CD_35_(-C)	5226783	5226784	NM_000518.4:c.108del
chr11	CD_35_TAC>TAA	5226783	5226784	NM_000518.4:c.108C>A
chr11	CD_33/34_-GTG_[-Val]	5226789	5226791	NM_000518.4:c.100_102delGTG
chr11	IVS_I-130_(G>A)	5226799	5226800	NM_000518.4:c.93-1G>A
chr11	IVS_I-130_G>C	5226799	5226800	NM_000518.4:c.93-1G>C
chr11	IVS_I-110_G>A	5226819	5226820	NM_000518.4:c.93-21G>A
chr11	IVS_I-6_(T>C)	5226923	5226924	NM_000518.4:c.92+6T>C
chr11	IVS_I-5_(G>A)	5226924	5226925	NM_000518.4:c.92+5G>A
chr11	IVS_I-5_(G>T)	5226924	5226925	NM_000518.4:c.92+5G>T
chr11	IVS_I-5_(G>C)	5226924	5226925	NM_000518.4:c.92+5G>C
chr11	IVS_I-2_(T>C)	5226927	5226928	NM_000518.4:c.92+2T>C
chr11	IVS_I-2_(T>G)	5226927	5226928	NM_000518.4:c.92+2T>G
chr11	IVS_I-1_(G>C)	5226928	5226929	NM_000518.4:c.92+1G>C
chr11	IVS_I-1_(G>T)	5226928	5226929	NM_000518.4:c.92+1G>T
chr11	IVS_I-1_G>A	5226928	5226929	NM_000518.4:c.92+1G>A
chr11	CD_30_(G>C)_or__IVS_I__(-1)_AGG>ACG_(Arg>Thr)	5226929	5226930	NM_000518.4:c.92G>C
chr11	CD_30_(A>G)_or__IVS_I__(-2)_AGG>GGG_(Arg>Gly)	5226930	5226931	NM_000518.4:c.91A>G
chr11	CD_29_(C>T)_or__IVS_I__(-3)_GGC>GGT_(Gly>Gly)	5226931	5226932	NM_000518.4:c.90C>T
chr11	CD_28_(CTG>CGG)_Leu_>Arg	5226935	5226936	NM_000518.4:c.86T>G
chr11	CD_27/28_(+C)	5226936	5226937	NM_000518.4:c.84_85insC
chr11	CD_27_GCC>TCC_[Ala>Ser]	5226939	5226940	NM_000518.4:c.82G>T
chr11	CD_26_GAG>AAG_[Glu>Lys]	5226942	5226943	NM_000518.4:c.79G>A

chr11	CD_24_GGT>GGA_[Gly>Gly]	5226946	5226947	NM_000518.4:c.75T>A
chr11	CD_19_(AAC>AGC)_[Asn>Ser]	5226962	5226963	NM_000518.4:c.59A>G
chr11	CD_17_AAG>ATG_(Lys>Met)	5226968	5226969	NM_000518.4:c.53A>T
chr11	CD_17_(AAG>TAG)	5226969	5226970	NM_000518.4:c.52A>T
chr11	CD_16_GGC>GG-	5226970	5226971	NM_000518.4:c.51delC
chr11	CD_15_TGG>TGA	5226973	5226974	NM_000518.4:c.48G>A
chr11	CD_15_TGG>TAG	5226974	5226975	NM_000518.4:c.47G>A
chr11	CD_8/9_+G	5226993	5226994	NM_000518.4:c.27_28insG
chr11	CD_8_(-AA)	5226995	5226996	NM_000518.4:c.25_26delAA
chr11	CD_6_-A	5227001	5227002	NM_000518.4:c.20delA
chr11	CD_6_GAG>GTG_[Glu>Val]	5227001	5227002	NM_000518.4:c.20A>T
chr11	CD_6_GAG>AAG_[Glu>Lys]	5227002	5227003	NM_000518.4:c.19G>A
chr11	CD_5_-CT	5227003	5227004	NM_000518.4:c.17_18delCT
chr11	Init_CD__ATG>ATA	5227018	5227019	NM_000518.4:c.3G>A
chr11	Init_CD__ATG>ACG	5227019	5227020	NM_000518.4:c.2T>C
chr11	Init_CD__ATG>AGG	5227019	5227020	NM_000518.4:c.2T>G
chr11	Init_CD__ATG>GTG	5227020	5227021	NM_000518.4:c.1A>G
chr11	CAP_+1_(A>C)	5227070	5227071	NM_000518.4:c.-50A>C
chr11	-28_(A>G)	5227098	5227099	NM_000518.4:c.-78A>G
chr11	-28_(A>C)	5227098	5227099	NM_000518.4:c.-78A>C
chr11	-29_(A>G)	5227099	5227100	NM_000518.4:c.-79A>G
chr11	-30_(T>A)	5227100	5227101	NM_000518.4:c.-80T>A
chr11	-31_(A>G)	5227101	5227102	NM_000518.4:c.-81A>G
chr11	-87_(C>T)	5227157	5227158	NM_000518.4:c.-137C>T
chr11	-87_C>G	5227157	5227158	NM_000518.4:c.-137C>G
chr11	-87_(C>A)	5227157	5227158	NM_000518.4:c.-137C>A
chr11	-88_(C>T)	5227158	5227159	NM_000518.4:c.-138C>T
chr11	-101_(C>T)	5227171	5227172	NM_000518.4:c.-151C>T

Table S3. Fraction of minor genotype in the spiked genomic DNA samples per informative SNV

spike_ID	*rs number*	*POS*	*Total Reads*	*REF*	*ALT*	*spike_REF Reads*	*spike_ALT Reads*	*GT in majority*	*GT in minority*	*Expected GT in spikes*	*Fraction of minor GT*
spike1.25%	rs6578588	5231021	11950	T	C	6	11944	CC	TC	TC	0.1
spike1.25%	rs7948668	5235417	6616	A	G	6	6610	GG	AG	AG	0.181
spike1.25%	rs3759074	5236548	11321	G	A	11241	80	GG	GA	GA	1.413
spike1.25%	rs10837643	5236808	6711	T	A	2	6709	AA	TA	TA	0.06
spike1.25%	rs4320977	5236932	9438	A	G	60	9378	GG	AG	AG	1.271
spike1.25%	rs3759073	5237035	8816	T	C	2	8814	CC	TC	TC	0.045
spike1.25%	rs4283007	5237260	11236	A	T	60	11176	TT	AT	AT	1.068
spike1.25%	rs4402323	5237362	13185	C	T	12	13173	TT	CT	CT	0.182
spike1.25%	rs4910543	5237597	13849	G	C	1	13848	CC	GC	GC	0.014
spike1.25%	rs4910735	5237622	13818	G	A	19	13799	AA	GA	GA	0.275
spike1.25%	rs4910544	5237626	13813	T	A	3	13810	AA	TA	TA	0.043
spike1.25%	rs4910736	5237759	10479	C	A	65	10414	AA	CA	CA	1.241
spike1.25%	rs2105819	5238497	8952	G	C	55	8897	CC	GC	GC	1.229
spike1.25%	rs968857	5239228	9168	T	C	4	9164	CC	TC	TC	0.087
spike1.25%	rs968856	5239346	9610	T	C	110	9500	CC	TC	TC	2.289
spike1.25%	rs10768687	5240009	11058	C	G	0	11058	GG	CG	CG	0
spike1.25%	rs10128556	5242453	12924	C	T	12909	15	CC	CT	CT	0.232
spike1.25%	rs2071348	5242916	9133	T	G	9016	117	TT	TG	TG	2.562
spike1.25%	rs12417960	5243876	10266	C	A	10161	105	CC	CA	CA	2.046
spike1.25%	rs7924684	5245498	7862	C	T	12	7850	TT	CT	CT	0.305

spike1.25%	rs7480197	5247176	12315	C	T	11	12304	TT	CT	CT	0.179
spike1.25%	rs7483789	5247392	13948	T	G	264	13684	GG	TG	TG	3.785
spike1.25%	rs10488676	5247567	16202	G	A	26	16176	AA	GA	GA	0.321
spike1.25%	rs916111	5248113	11834	T	A	2	11832	AA	TA	TA	0.034
spike1.25%	rs2187608	5248701	11317	G	C	11316	1	GG	GC	GC	0.018
spike1.25%	rs2855039	5250441	13215	C	T	13190	25	CC	CT	CT	0.378
spike1.25%	rs2855038	5250924	14963	T	C	150	14813	CC	TC	TC	2.005
spike1.25%	rs2855036	5251452	15949	C	T	15814	135	CC	CT	CT	1.693
spike1.25%	rs2855126	5251917	13917	C	G	90	13827	GG	CG	CG	1.293
spike1.25%	rs2255519	5252311	11527	G	A	20	11507	AA	GA	GA	0.347
spike1.25%	rs2855125	5252457	14070	T	G	123	13947	GG	TG	TG	1.748
spike1.25%	rs2236794	5253037	12773	C	T	148	12625	TT	CT	CT	2.317
spike1.25%	rs2011051	5255588	10750	G	T	2	10748	TT	GT	GT	0.037
spike1.25%	rs2855123	5255848	11899	A	T	125	11774	TT	AT	AT	2.101
spike1.25%	rs2855122	5256006	11647	C	T	22	11625	TT	CT	CT	0.378
spike1.25%	rs2855121	5256061	25627	C	T	25482	145	CC	CT	CT	1.132
spike1.25%	rs11036496	5258792	12969	G	C	129	12840	CC	GC	GC	1.989
spike1.25%	rs12284340	5259051	6504	C	T	6495	9	CC	CT	CT	0.277
spike1.25%	rs10837697	5259788	11182	G	C	11182	0	GG	GC	GC	0
spike1.25%	rs10160820	5261227	9587	A	C	9586	1	AA	AC	AC	0.021
spike1.25%	rs7480802	5261278	9639	T	C	6	9633	CC	TC	TC	0.124
spike1.25%	rs11036506	5262292	10112	A	G	10011	101	AA	AG	AG	1.998
spike1.25%	rs4348933	5263748	12282	A	G	9	12273	GG	AG	AG	0.147
spike1.25%	rs4910546	5265082	11280	T	C	23	11257	CC	TC	TC	0.408
spike1.25%	rs10837707	5265578	10544	T	C	106	10438	CC	TC	TC	2.011
spike1.25%	rs4910740	5266060	8224	G	A	101	8123	AA	GA	GA	2.456
spike1.25%	rs72872548	5267909	10504	C	A	10385	119	CC	CA	CA	2.266

spike1.25%	rs72872549	5268823	12454	C	T	12301	153	CC	CT	CT	2.457
spike1.25%	rs67385638	5269140	13638	C	G	13638	0	CC	CG	CG	0
spike1.25%	rs3759071	5270302	13406	G	A	89	13317	AA	GA	GA	1.328
spike1.25%	rs3759070	5270398	12120	C	G	12120	0	CC	CG	CG	0
spike1.25%	rs12295181	5270506	13850	C	T	13708	142	CC	CT	CT	2.051
spike1.25%	rs3759069	5270600	21889	A	G	12	21877	GG	AG	AG	0.11
spike1.25%	rs10768737	5270642	29742	T	C	73	29669	CC	TC	TC	0.491
spike1.25%	rs11036562	5272915	8595	C	A	7	8588	AA	CA	CA	0.163
spike1.25%	rs10488675	5276800	13531	A	G	16	13515	GG	AG	AG	0.236
spike1.25%	rs11036634	5287498	2728	C	T	3	2725	TT	CT	CT	0.22
spike1.25%	rs11036635	5287666	12050	G	A	12046	4	GG	GA	GA	0.066
spike1.25%	rs7119142	5287848	15930	A	G	15909	21	AA	AG	AG	0.264
spike1.25%	rs16912979	5288465	11934	T	C	11932	2	TT	TC	TC	0.034
spike1.25%	rs4910743	5288920	14112	T	C	12	14100	CC	TC	TC	0.17
spike1.25%	rs72872555	5288939	14106	G	A	14103	3	GG	GA	GA	0.043
spike1.25%	rs11036639	5289276	11810	A	C	2	11808	CC	AC	AC	0.034
spike1.25%	rs11036641	5289348	13260	A	G	13253	7	AA	AG	AG	0.106
spike1.25%	rs11036644	5289833	10138	A	T	2	10136	TT	AT	AT	0.039
spike1.25%	rs4601817	5291171	11536	A	G	157	11379	GG	AG	AG	2.722
spike1.25%	rs3888708	5297150	9584	C	A	99	9485	AA	CA	CA	2.066
spike 2.5%	rs6578588	5231021	12907	T	C	113	12794	CC	TC	TC	1.751
spike 2.5%	rs7948668	5235417	7437	A	G	54	7383	GG	AG	AG	1.452
spike 2.5%	rs3759074	5236548	9613	G	A	9605	8	GG	GA	GA	0.166
spike 2.5%	rs10837643	5236808	7664	T	A	73	7591	AA	TA	TA	1.905
spike 2.5%	rs4320977	5236932	8023	A	G	69	7954	GG	AG	AG	1.72
spike 2.5%	rs3759073	5237035	9607	T	C	112	9495	CC	TC	TC	2.332
spike 2.5%	rs4283007	5237260	10120	A	T	100	10020	TT	AT	AT	1.976

spike 2.5%	rs4402323	5237362	13648	C	T	162	13486	TT	CT	CT	2.374
spike 2.5%	rs4910543	5237597	13975	G	C	153	13822	CC	GC	GC	2.19
spike 2.5%	rs4910735	5237622	13952	G	A	164	13788	AA	GA	GA	2.351
spike 2.5%	rs4910544	5237626	13942	T	A	155	13787	AA	TA	TA	2.223
spike 2.5%	rs4910736	5237759	8810	C	A	98	8712	AA	CA	CA	2.225
spike 2.5%	rs2105819	5238497	8561	G	C	107	8454	CC	GC	GC	2.5
spike 2.5%	rs968857	5239228	10453	T	C	124	10329	CC	TC	TC	2.373
spike 2.5%	rs968856	5239346	9446	T	C	125	9321	CC	TC	TC	2.647
spike 2.5%	rs10768687	5240009	11669	C	G	123	11546	GG	CG	CG	2.108
spike 2.5%	rs10128556	5242453	14794	C	T	14652	142	CC	CT	CT	1.92
spike 2.5%	rs2071348	5242916	11564	T	G	11456	108	TT	TG	TG	1.868
spike 2.5%	rs12417960	5243876	11110	C	A	10988	122	CC	CA	CA	2.196
spike 2.5%	rs7924684	5245498	7717	C	T	96	7621	TT	CT	CT	2.488
spike 2.5%	rs7480197	5247176	10117	C	T	146	9971	TT	CT	CT	2.886
spike 2.5%	rs7483789	5247392	13195	T	G	241	12954	GG	TG	TG	3.653
spike 2.5%	rs10488676	5247567	12403	G	A	191	12212	AA	GA	GA	3.08
spike 2.5%	rs916111	5248113	9233	T	A	125	9108	AA	TA	TA	2.708
spike 2.5%	rs2187608	5248701	9226	G	C	9101	125	GG	GC	GC	2.71
spike 2.5%	rs2855039	5250441	12218	C	T	12070	148	CC	CT	CT	2.423
spike 2.5%	rs2855038	5250924	12983	T	C	13	12970	CC	TC	TC	0.2
spike 2.5%	rs2855036	5251452	13290	C	T	13281	9	CC	CT	CT	0.135
spike 2.5%	rs2855126	5251917	11651	C	G	2	11649	GG	CG	CG	0.034
spike 2.5%	rs2255519	5252311	10859	G	A	143	10716	AA	GA	GA	2.634
spike 2.5%	rs2855125	5252457	11432	T	G	3	11429	GG	TG	TG	0.052
spike 2.5%	rs2236794	5253037	12019	C	T	32	11987	TT	CT	CT	0.532
spike 2.5%	rs2011051	5255588	10197	G	T	0	10197	TT	GT	GT	0
spike 2.5%	rs2855123	5255848	11922	A	T	4	11918	TT	AT	AT	0.067

spike 2.5%	rs2855122	5256006	11089	C	T	25	11064	TT	CT	CT	0.451
spike 2.5%	rs2855121	5256061	24550	C	T	24485	65	CC	CT	CT	0.53
spike 2.5%	rs11036496	5258792	13430	G	C	2	13428	CC	GC	GC	0.03
spike 2.5%	rs12284340	5259051	5393	C	T	5385	8	CC	CT	CT	0.297
spike 2.5%	rs10837697	5259788	10017	G	C	10015	2	GG	GC	GC	0.04
spike 2.5%	rs10160820	5261227	9883	A	C	9881	2	AA	AC	AC	0.04
spike 2.5%	rs7480802	5261278	9924	T	C	7	9917	CC	TC	TC	0.141
spike 2.5%	rs11036506	5262292	9522	A	G	9503	19	AA	AG	AG	0.399
spike 2.5%	rs4348933	5263748	12335	A	G	6	12329	GG	AG	AG	0.097
spike 2.5%	rs4910546	5265082	10682	T	C	12	10670	CC	TC	TC	0.225
spike 2.5%	rs10837707	5265578	10772	T	C	3	10769	CC	TC	TC	0.056
spike 2.5%	rs4910740	5266060	9089	G	A	5	9084	AA	GA	GA	0.11
spike 2.5%	rs72872548	5267909	11467	C	A	11467	0	CC	CA	CA	0
spike 2.5%	rs72872549	5268823	14701	C	T	14694	7	CC	CT	CT	0.095
spike 2.5%	rs67385638	5269140	13416	C	G	13282	134	CC	CG	CG	1.998
spike 2.5%	rs3759071	5270302	15059	G	A	25	15034	AA	GA	GA	0.332
spike 2.5%	rs3759070	5270398	11894	C	G	11794	100	CC	CG	CG	1.682
spike 2.5%	rs12295181	5270506	18703	C	T	18691	12	CC	CT	CT	0.128
spike 2.5%	rs3759069	5270600	20501	A	G	159	20342	GG	AG	AG	1.551
spike 2.5%	rs10768737	5270642	29665	T	C	172	29493	CC	TC	TC	1.16
spike 2.5%	rs11036562	5272915	7509	C	A	93	7416	AA	CA	CA	2.477
spike 2.5%	rs10488675	5276800	11426	A	G	12	11414	GG	AG	AG	0.21
spike 2.5%	rs11036634	5287498	2926	C	T	88	2838	TT	CT	CT	6.015
spike 2.5%	rs11036635	5287666	11714	G	A	11425	289	GG	GA	GA	4.934
spike 2.5%	rs7119142	5287848	18215	A	G	17654	561	AA	AG	AG	6.16
spike 2.5%	rs16912979	5288465	11775	T	C	11544	231	TT	TC	TC	3.924
spike 2.5%	rs4910743	5288920	14851	T	C	456	14395	CC	TC	TC	6.141

spike 2.5%	rs72872555	5288939	14830	G	A	14385	445	GG	GA	GA	6.001
spike 2.5%	rs11036639	5289276	10899	A	C	261	10638	CC	AC	AC	4.789
spike 2.5%	rs11036641	5289348	13810	A	G	13329	481	AA	AG	AG	6.966
spike 2.5%	rs11036644	5289833	10511	A	T	205	10306	TT	AT	AT	3.901
spike 2.5%	rs4601817	5291171	12881	A	G	254	12627	GG	AG	AG	3.944
spike 2.5%	rs3888708	5297150	10487	C	A	293	10194	AA	CA	CA	5.588
spike 5%	rs6578588	5231021	10548	T	C	198	10350	CC	TC	TC	3.754
spike 5%	rs7948668	5235417	7142	A	G	70	7072	GG	AG	AG	1.96
spike 5%	rs3759074	5236548	10940	G	A	10837	103	GG	GA	GA	1.883
spike 5%	rs10837643	5236808	6580	T	A	97	6483	AA	TA	TA	2.948
spike 5%	rs4320977	5236932	9717	A	G	99	9618	GG	AG	AG	2.038
spike 5%	rs3759073	5237035	8212	T	C	98	8114	CC	TC	TC	2.387
spike 5%	rs4283007	5237260	11156	A	T	85	11071	TT	AT	AT	1.524
spike 5%	rs4402323	5237362	12515	C	T	143	12372	TT	CT	CT	2.285
spike 5%	rs4910543	5237597	12115	G	C	193	11922	CC	GC	GC	3.186
spike 5%	rs4910735	5237622	12101	G	A	220	11881	AA	GA	GA	3.636
spike 5%	rs4910544	5237626	12080	T	A	195	11885	AA	TA	TA	3.228
spike 5%	rs4910736	5237759	11018	C	A	131	10887	AA	CA	CA	2.378
spike 5%	rs2105819	5238497	11328	G	C	123	11205	CC	GC	GC	2.172
spike 5%	rs968857	5239228	10064	T	C	61	10003	CC	TC	TC	1.212
spike 5%	rs968856	5239346	11645	T	C	113	11532	CC	TC	TC	1.941
spike 5%	rs10768687	5240009	11286	C	G	63	11223	GG	CG	CG	1.116
spike 5%	rs10128556	5242453	11727	C	T	11400	327	CC	CT	CT	5.577
spike 5%	rs2071348	5242916	12007	T	G	11929	78	TT	TG	TG	1.299
spike 5%	rs12417960	5243876	11784	C	A	11684	100	CC	CA	CA	1.697
spike 5%	rs7924684	5245498	6928	C	T	85	6843	TT	CT	CT	2.454
spike 5%	rs7480197	5247176	11766	C	T	140	11626	TT	CT	CT	2.38

spike 5%	rs7483789	5247392	15532	T	G	246	15286	GG	TG	TG	3.168
spike 5%	rs10488676	5247567	13309	G	A	167	13142	AA	GA	GA	2.51
spike 5%	rs916111	5248113	10920	T	A	132	10788	AA	TA	TA	2.418
spike 5%	rs2187608	5248701	10721	G	C	10609	112	GG	GC	GC	2.089
spike 5%	rs2855039	5250441	12972	C	T	12958	14	CC	CT	CT	0.216
spike 5%	rs2855038	5250924	13638	T	C	283	13355	CC	TC	TC	4.15
spike 5%	rs2855036	5251452	14809	C	T	14523	286	CC	CT	CT	3.863
spike 5%	rs2855126	5251917	12548	C	G	234	12314	GG	CG	CG	3.73
spike 5%	rs2255519	5252311	12325	G	A	204	12121	AA	GA	GA	3.31
spike 5%	rs2855125	5252457	13243	T	G	246	12997	GG	TG	TG	3.715
spike 5%	rs2236794	5253037	13931	C	T	231	13700	TT	CT	CT	3.316
spike 5%	rs2011051	5255588	9820	G	T	127	9693	TT	GT	GT	2.587
spike 5%	rs2855123	5255848	14068	A	T	356	13712	TT	AT	AT	5.061
spike 5%	rs2855122	5256006	11754	C	T	109	11645	TT	CT	CT	1.855
spike 5%	rs2855121	5256061	26941	C	T	26482	459	CC	CT	CT	3.407
spike 5%	rs11036496	5258792	14259	G	C	481	13778	CC	GC	GC	6.747
spike 5%	rs12284340	5259051	5868	C	T	5804	64	CC	CT	CT	2.181
spike 5%	rs10837697	5259788	10157	G	C	10077	80	GG	GC	GC	1.575
spike 5%	rs10160820	5261227	9532	A	C	9531	1	AA	AC	AC	0.021
spike 5%	rs7480802	5261278	9583	T	C	10	9573	CC	TC	TC	0.209
spike 5%	rs11036506	5262292	10761	A	G	10622	139	AA	AG	AG	2.583
spike 5%	rs4348933	5263748	12713	A	G	136	12577	GG	AG	AG	2.14
spike 5%	rs4910546	5265082	11196	T	C	267	10929	CC	TC	TC	4.77
spike 5%	rs10837707	5265578	11017	T	C	362	10655	CC	TC	TC	6.572
spike 5%	rs4910740	5266060	8755	G	A	270	8485	AA	GA	GA	6.168
spike 5%	rs72872548	5267909	11015	C	A	10776	239	CC	CA	CA	4.34
spike 5%	rs72872549	5268823	13408	C	T	13183	225	CC	CT	CT	3.356

spike 5%	rs67385638	5269140	14199	C	G	14121	78	CC	CG	CG	1.099
spike 5%	rs3759071	5270302	13710	G	A	324	13386	AA	GA	GA	4.726
spike 5%	rs3759070	5270398	11022	C	G	10947	75	CC	CG	CG	1.361
spike 5%	rs12295181	5270506	16812	C	T	16090	722	CC	CT	CT	8.589
spike 5%	rs3759069	5270600	18652	A	G	289	18363	GG	AG	AG	3.099
spike 5%	rs10768737	5270642	25784	T	C	529	25255	CC	TC	TC	4.103
spike 5%	rs11036562	5272915	7990	C	A	109	7881	AA	CA	CA	2.728
spike 5%	rs10488675	5276800	12822	A	G	287	12535	GG	AG	AG	4.477
spike 5%	rs11036634	5287498	2560	C	T	32	2528	TT	CT	CT	2.5
spike 5%	rs11036635	5287666	13625	G	A	13376	249	GG	GA	GA	3.655
spike 5%	rs7119142	5287848	14986	A	G	14644	342	AA	AG	AG	4.564
spike 5%	rs16912979	5288465	12726	T	C	12443	283	TT	TC	TC	4.448
spike 5%	rs4910743	5288920	11658	T	C	53	11605	CC	TC	TC	0.909
spike 5%	rs72872555	5288939	11654	G	A	11603	51	GG	GA	GA	0.875
spike 5%	rs11036639	5289276	11300	A	C	240	11060	CC	AC	AC	4.248
spike 5%	rs11036641	5289348	11606	A	G	11499	107	AA	AG	AG	1.844
spike 5%	rs11036644	5289833	9544	A	T	194	9350	TT	AT	AT	4.065
spike 5%	rs4601817	5291171	12412	A	G	118	12294	GG	AG	AG	1.901
spike 5%	rs3888708	5297150	10282	C	A	71	10211	AA	CA	CA	1.381
spike 10%	rs6578588	5231021	11815	T	C	566	11249	CC	TC	TC	9.581
spike 10%	rs7948668	5235417	7461	A	G	155	7306	GG	AG	AG	4.155
spike 10%	rs3759074	5236548	10758	G	A	10401	357	GG	GA	GA	6.637
spike 10%	rs10837643	5236808	7523	T	A	132	7391	AA	TA	TA	3.509
spike 10%	rs4320977	5236932	9480	A	G	428	9052	GG	AG	AG	9.03
spike 10%	rs3759073	5237035	9400	T	C	201	9199	CC	TC	TC	4.277
spike 10%	rs4283007	5237260	11274	A	T	405	10869	TT	AT	AT	7.185
spike 10%	rs4402323	5237362	14989	C	T	323	14666	TT	CT	CT	4.31

spike 10%	rs4910543	5237597	14482	G	C	464	14018	CC	GC	GC	6.408
spike 10%	rs4910735	5237622	14456	G	A	483	13973	AA	GA	GA	6.682
spike 10%	rs4910544	5237626	14450	T	A	466	13984	AA	TA	TA	6.45
spike 10%	rs4910736	5237759	9879	C	A	419	9460	AA	CA	CA	8.483
spike 10%	rs2105819	5238497	10198	G	C	497	9701	CC	GC	GC	9.747
spike 10%	rs968857	5239228	11538	T	C	476	11062	CC	TC	TC	8.251
spike 10%	rs968856	5239346	11214	T	C	795	10419	CC	TC	TC	14.179
spike 10%	rs10768687	5240009	13474	C	G	408	13066	GG	CG	CG	6.056
spike 10%	rs10128556	5242453	14755	C	T	14139	616	CC	CT	CT	8.35
spike 10%	rs2071348	5242916	9896	T	G	9592	304	TT	TG	TG	6.144
spike 10%	rs12417960	5243876	9410	C	A	9128	282	CC	CA	CA	5.994
spike 10%	rs7924684	5245498	7449	C	T	305	7144	TT	CT	CT	8.189
spike 10%	rs7480197	5247176	11281	C	T	334	10947	TT	CT	CT	5.921
spike 10%	rs7483789	5247392	13164	T	G	591	12573	GG	TG	TG	8.979
spike 10%	rs10488676	5247567	13667	G	A	407	13260	AA	GA	GA	5.956
spike 10%	rs916111	5248113	10280	T	A	432	9848	AA	TA	TA	8.405
spike 10%	rs2187608	5248701	9499	G	C	9056	443	GG	GC	GC	9.327
spike 10%	rs2855039	5250441	13876	C	T	13227	649	CC	CT	CT	9.354
spike 10%	rs2855038	5250924	11976	T	C	519	11457	CC	TC	TC	8.667
spike 10%	rs2855036	5251452	12323	C	T	11706	617	CC	CT	CT	10.014
spike 10%	rs2855126	5251917	10829	C	G	574	10255	GG	CG	CG	10.601
spike 10%	rs2255519	5252311	12368	G	A	488	11880	AA	GA	GA	7.891
spike 10%	rs2855125	5252457	10723	T	G	475	10248	GG	TG	TG	8.859
spike 10%	rs2236794	5253037	11234	C	T	478	10756	TT	CT	CT	8.51
spike 10%	rs2011051	5255588	11669	G	T	360	11309	TT	GT	GT	6.17
spike 10%	rs2855123	5255848	11322	A	T	512	10810	TT	AT	AT	9.044
spike 10%	rs2855122	5256006	12657	C	T	456	12201	TT	CT	CT	7.205

spike 10%	rs2855121	5256061	25909	C	T	24828	1081	CC	CT	CT	8.345
spike 10%	rs11036496	5258792	12895	G	C	756	12139	CC	GC	GC	11.725
spike 10%	rs12284340	5259051	6016	C	T	5696	320	CC	CT	CT	10.638
spike 10%	rs10837697	5259788	12773	G	C	12262	511	GG	GC	GC	8.001
spike 10%	rs10160820	5261227	9566	A	C	9043	523	AA	AC	AC	10.935
spike 10%	rs7480802	5261278	9610	T	C	523	9087	CC	TC	TC	10.884
spike 10%	rs11036506	5262292	9818	A	G	9428	390	AA	AG	AG	7.945
spike 10%	rs4348933	5263748	13242	A	G	536	12706	GG	AG	AG	8.095
spike 10%	rs4910546	5265082	13802	T	C	591	13211	CC	TC	TC	8.564
spike 10%	rs10837707	5265578	10014	T	C	767	9247	CC	TC	TC	15.319
spike 10%	rs4910740	5266060	8457	G	A	603	7854	AA	GA	GA	14.26
spike 10%	rs72872548	5267909	9557	C	A	8747	810	CC	CA	CA	16.951
spike 10%	rs72872549	5268823	11631	C	T	10913	718	CC	CT	CT	12.346
spike 10%	rs67385638	5269140	14615	C	G	14217	398	CC	CG	CG	5.446
spike 10%	rs3759071	5270302	13005	G	A	788	12217	AA	GA	GA	12.118
spike 10%	rs3759070	5270398	9755	C	G	9464	291	CC	CG	CG	5.966
spike 10%	rs12295181	5270506	18844	C	T	17252	1592	CC	CT	CT	16.897
spike 10%	rs3759069	5270600	18908	A	G	523	18385	GG	AG	AG	5.532
spike 10%	rs10768737	5270642	26845	T	C	1079	25766	CC	TC	TC	8.039
spike 10%	rs11036562	5272915	9484	C	A	387	9097	AA	CA	CA	8.161
spike 10%	rs10488675	5276800	12449	A	G	228	12221	GG	AG	AG	3.663
spike 10%	rs11036634	5287498	1812	C	T	63	1749	TT	CT	CT	6.954
spike 10%	rs11036635	5287666	12695	G	A	12200	495	GG	GA	GA	7.798
spike 10%	rs7119142	5287848	12525	A	G	11955	570	AA	AG	AG	9.102
spike 10%	rs16912979	5288465	13432	T	C	12940	492	TT	TC	TC	7.326
spike 10%	rs4910743	5288920	10075	T	C	487	9588	CC	TC	TC	9.667
spike 10%	rs72872555	5288939	10074	G	A	9595	479	GG	GA	GA	9.51

spike 10%	rs11036639	5289276	12883	A	C	475	12408	CC	AC	AC	7.374
spike 10%	rs11036641	5289348	10693	A	G	10197	496	AA	AG	AG	9.277
spike 10%	rs11036644	5289833	12356	A	T	439	11917	TT	AT	AT	7.106
spike 10%	rs4601817	5291171	15194	A	G	500	14694	GG	AG	AG	6.582
spike 10%	rs3888708	5297150	9928	C	A	677	9251	AA	CA	CA	13.638

Pos: Position, REF: Reference, ALT: Alternate, GT: Genotype

Table S5. Informative SNVs for paternally-inherited fetal allele detection where the mother is homozygous and the father is heterozygous

SNV	*chr*	*Position (GRCh38)*	*rs number*	*DOH (%)*
1	chr11	5218633	rs139797435	32.29
2	chr11	5222527	rs7930833	18.75
3	chr11	5222914	rs12364872	38.54
4	chr11	5223174	rs10837628	32.29
5	chr11	5224176	rs2187610	37.50
6	chr11	5224277	rs10768682	18.75
7	chr11	5224770	rs11036351	39.58
8	chr11	5224812	rs10837630	18.75
9	chr11	5224973	rs78928216	39.58
10	chr11	5225126	rs10837631	33.33
11	chr11	5225282	rs7110263	18.75
12	chr11	5225365	rs12788013	17.71
13	chr11	5225911	rs1609812	17.71
14	chr11	5226496	rs7946748	17.71
15	chr11	5226503	rs7480526	39.58
16	chr11	5226561	rs10768683	17.71
17	chr11	5227013	rs713040	17.71
18	chr11	5227411	rs10742583	18.75
19	chr11	5227599	rs74234654	41.66
20	chr11	5227774	rs11036364	55.21
21	chr11	5228140	rs1003586	32.29
22	chr11	5228938	rs7936823	55.21
23	chr11	5231021	rs6578588	51.04
24	chr11	5232247	rs12222763	57.29
25	chr11	5234682	rs3813727	57.29
26	chr11	5235417	rs7948668	52.08
27	chr11	5236548	rs3759074	34.38
28	chr11	5236808	rs10837643	51.04
29	chr11	5236932	rs4320977	51.04
30	chr11	5237035	rs3759073	51.04

31	chr11	5237260	rs4283007	48.96
32	chr11	5237362	rs4402323	51.04
33	chr11	5237463	rs72869882	16.67
34	chr11	5237597	rs4910543	51.04
35	chr11	5237622	rs4910735	51.04
36	chr11	5237626	rs4910544	51.04
37	chr11	5237759	rs4910736	51.04
38	chr11	5238497	rs2105819	51.04
39	chr11	5239228	rs968857	51.04
40	chr11	5239346	rs968856	51.04
41	chr11	5240009	rs10768687	51.04
42	chr11	5241552	rs11036415	26.04
43	chr11	5242347	rs10128555	26.04
44	chr11	5242453	rs10128556	33.33
45	chr11	5242916	rs2071348	33.33
46	chr11	5243699	rs7934275	26.04
47	chr11	5243876	rs12417960	34.38
48	chr11	5245498	rs7924684	52.08
49	chr11	5245731	rs6578591	28.13
50	chr11	5247176	rs7480197	53.13
51	chr11	5247392	rs7483789	53.13
52	chr11	5247567	rs10488676	53.13
53	chr11	5247910	rs6578592	29.17
54	chr11	5248113	rs916111	51.04
55	chr11	5248354	rs1065686	29.17
56	chr11	5248356	rs5789383	29.17
57	chr11	5248701	rs2187608	32.29
58	chr11	5250441	rs2855039	34.38
59	chr11	5250924	rs2855038	51.04
60	chr11	5251452	rs2855036	32.29
61	chr11	5251917	rs2855126	51.04
62	chr11	5252311	rs2255519	50.00
63	chr11	5252457	rs2855125	50.00
64	chr11	5252692	rs10768707	29.17
65	chr11	5253037	rs2236794	50.00
66	chr11	5253948	rs11036474	34.37
67	chr11	5254010	rs11036475	51.04
68	chr11	5255588	rs2011051	50.00

69	chr11	5255848	rs2855123	50.00
70	chr11	5256006	rs2855122	47.92
71	chr11	5256061	rs2855121	33.33
72	chr11	5257923	rs5010979	29.17
73	chr11	5258097	rs5010980	29.17
74	chr11	5258125	rs5010981	29.17
75	chr11	5258258	rs5010984	29.17
76	chr11	5258792	rs11036496	48.96
77	chr11	5259051	rs12284340	30.21
78	chr11	5259788	rs10837697	30.21
79	chr11	5260304	rs10734494	29.17
80	chr11	5261227	rs10160820	32.29
81	chr11	5261278	rs7480802	46.88
82	chr11	5261664	rs10160678	29.17
83	chr11	5262292	rs11036506	13.54
84	chr11	5263748	rs4348933	48.96
85	chr11	5264726	rs112857819	12.50
86	chr11	5265082	rs4910546	48.96
87	chr11	5265578	rs10837707	48.96
88	chr11	5265926	rs10768733	29.17
89	chr11	5266060	rs4910740	51.04
90	chr11	5266705	rs7481986	50
91	chr11	5267909	rs72872548	29.17
92	chr11	5268076	rs11822578	29.17
93	chr11	5268823	rs72872549	31.25
94	chr11	5269140	rs67385638	31.25
95	chr11	5270302	rs3759071	50.00
96	chr11	5270398	rs3759070	36.46
97	chr11	5270506	rs12295181	14.58
98	chr11	5270600	rs3759069	50.00
99	chr11	5270642	rs10768737	50.00
100	chr11	5270962	rs3759067	28.13
101	chr11	5271883	rs7479652	28.13
102	chr11	5272890	rs4910547	29.17
103	chr11	5272915	rs11036562	50.00
104	chr11	5273171	rs4910548	29.17
105	chr11	5273948	rs6578595	29.16
106	chr11	5274401	rs6578596	33.33

107	chr11	5274874	rs7130110	33.33
108	chr11	5276800	rs10488675	41.67
109	chr11	5287498	rs11036634	46.88
110	chr11	5287666	rs11036635	30.21
111	chr11	5287848	rs7119142	31.25
112	chr11	5288465	rs16912979	31.25
113	chr11	5288920	rs4910743	47.37
114	chr11	5288939	rs4910743	47.37
115	chr11	5289276	rs11036639	46.32
116	chr11	5289348	rs11036641	32.29
117	chr11	5289803	rs10768774	29.47
118	chr11	5289833	rs11036644	49.47
119	chr11	5290120	rs61893113	28.12
120	chr11	5291171	rs4601817	49.47
121	chr11	5291631	rs977711420	11.46
122	chr11	5293129	rs72872557	27.08
123	chr11	5295501	rs7933082	47.37
124	chr11	5296323	rs10837767	46.88
125	chr11	5297150	rs3888708	47.92

SNV: Single Nucleotide Variant, DOH: Degree Of Heterozygosity
